# Supplementary material for: Examination of the enrichment of neuronal extracellular vesicles from cell conditioned media and human plasma using an anti-NCAM immunocapture bead approach
Source: bioRxiv. 2025 May 14:2025.05.13.653678. Preprint. [Version 1] doi: 10.1101/2025.05.13.653678 (PMC12132510; doi:10.1101/2025.05.13.653678)
Supplement: Supplement 2 [file media-2.pptx]

## Slide 1
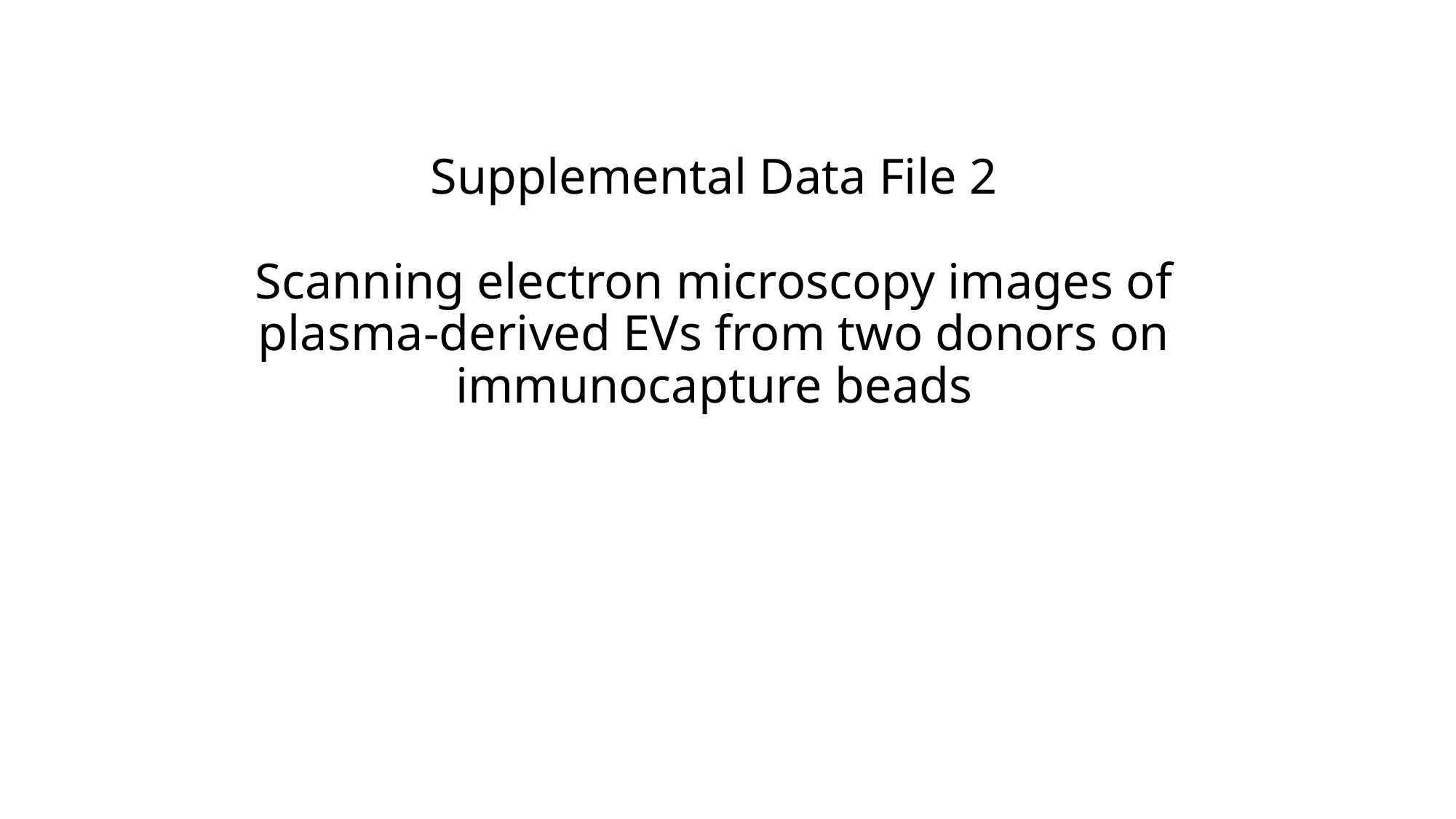

# Supplemental Data File 2 Scanning electron microscopy images of plasma-derived EVs from two donors on immunocapture beads

## Slide 2
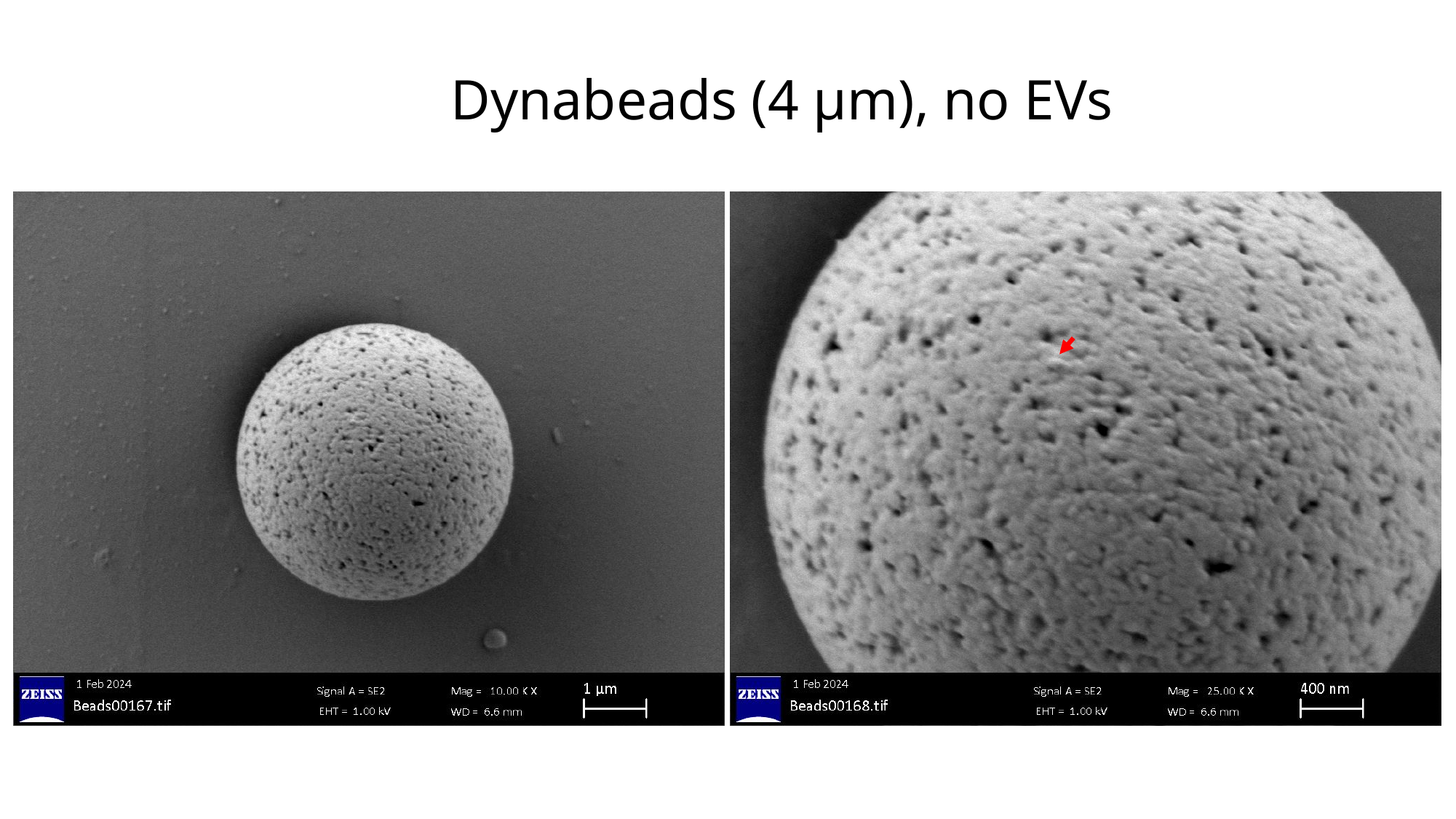

# Dynabeads (4 µm), no EVs

## Slide 3
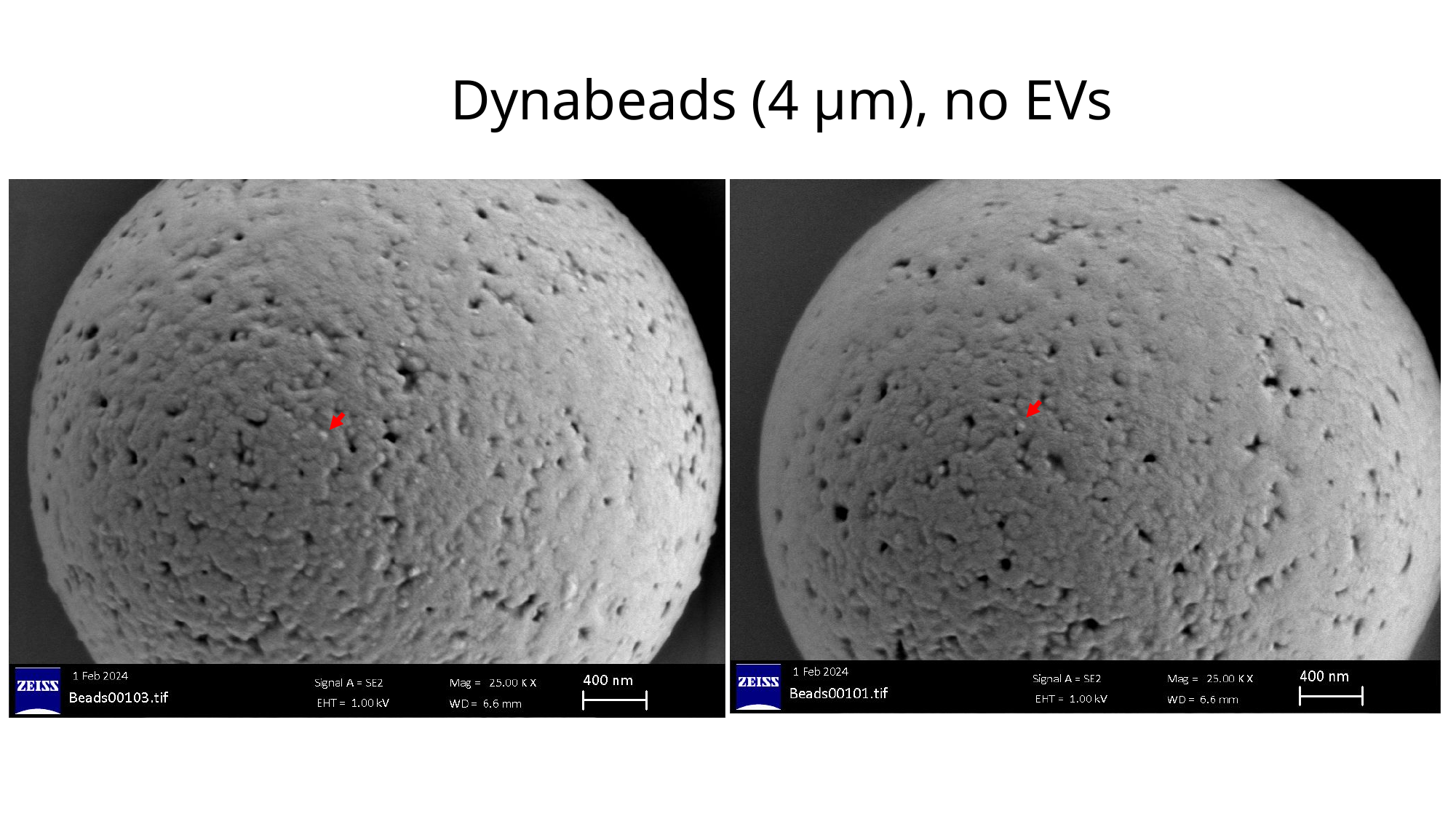

# Dynabeads (4 µm), no EVs

## Slide 4
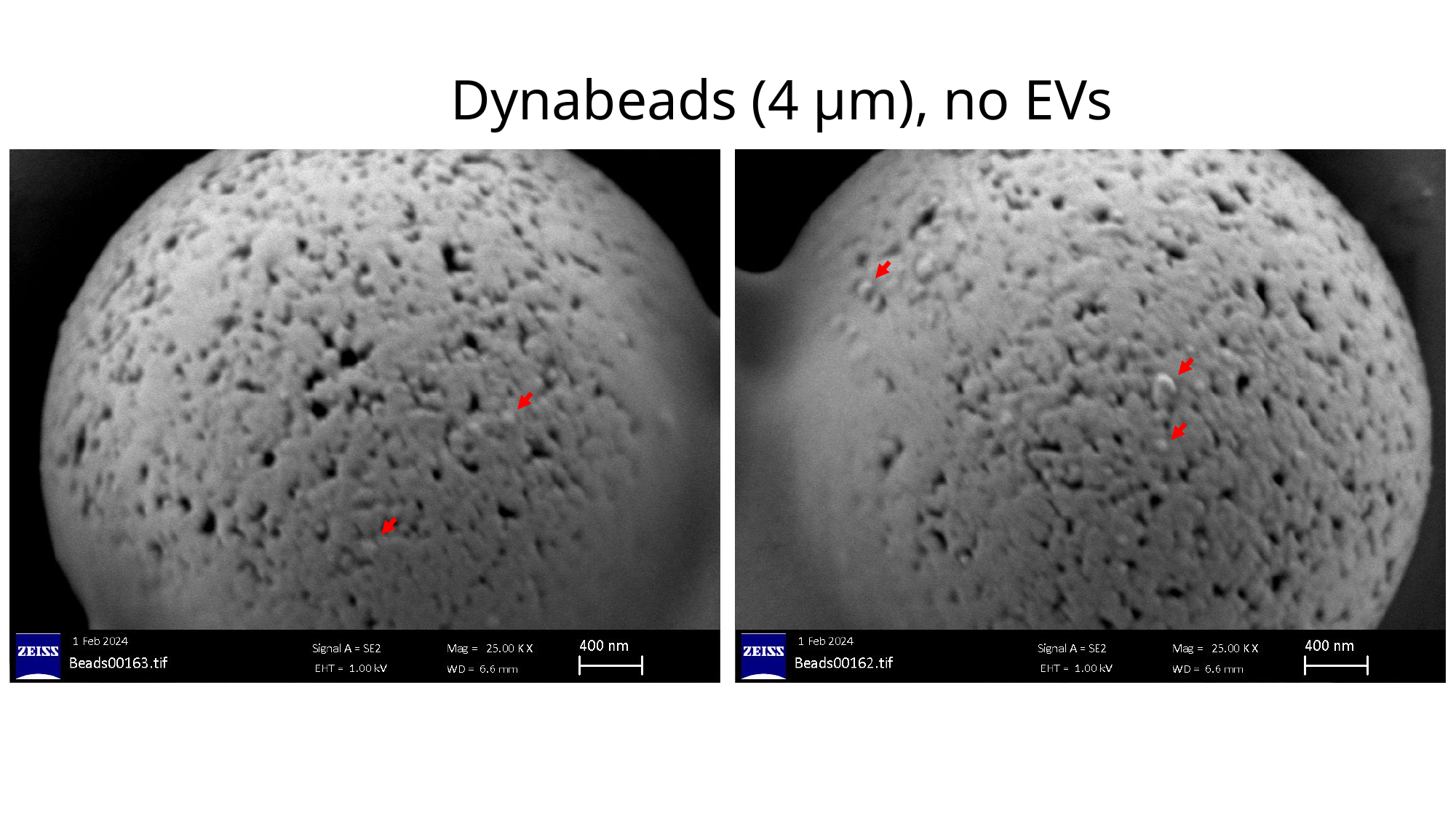

# Dynabeads (4 µm), no EVs

## Slide 5
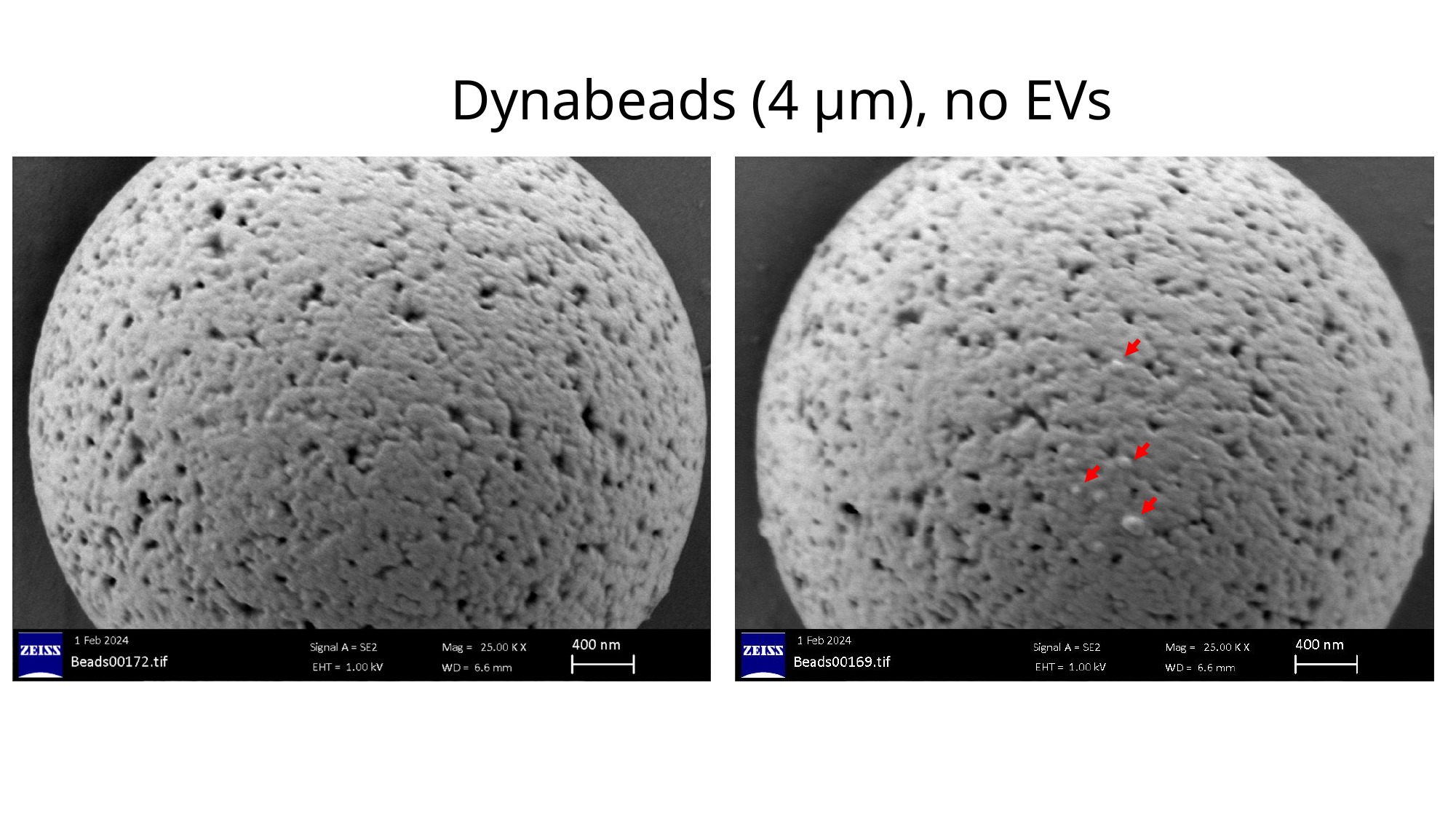

# Dynabeads (4 µm), no EVs

## Slide 6
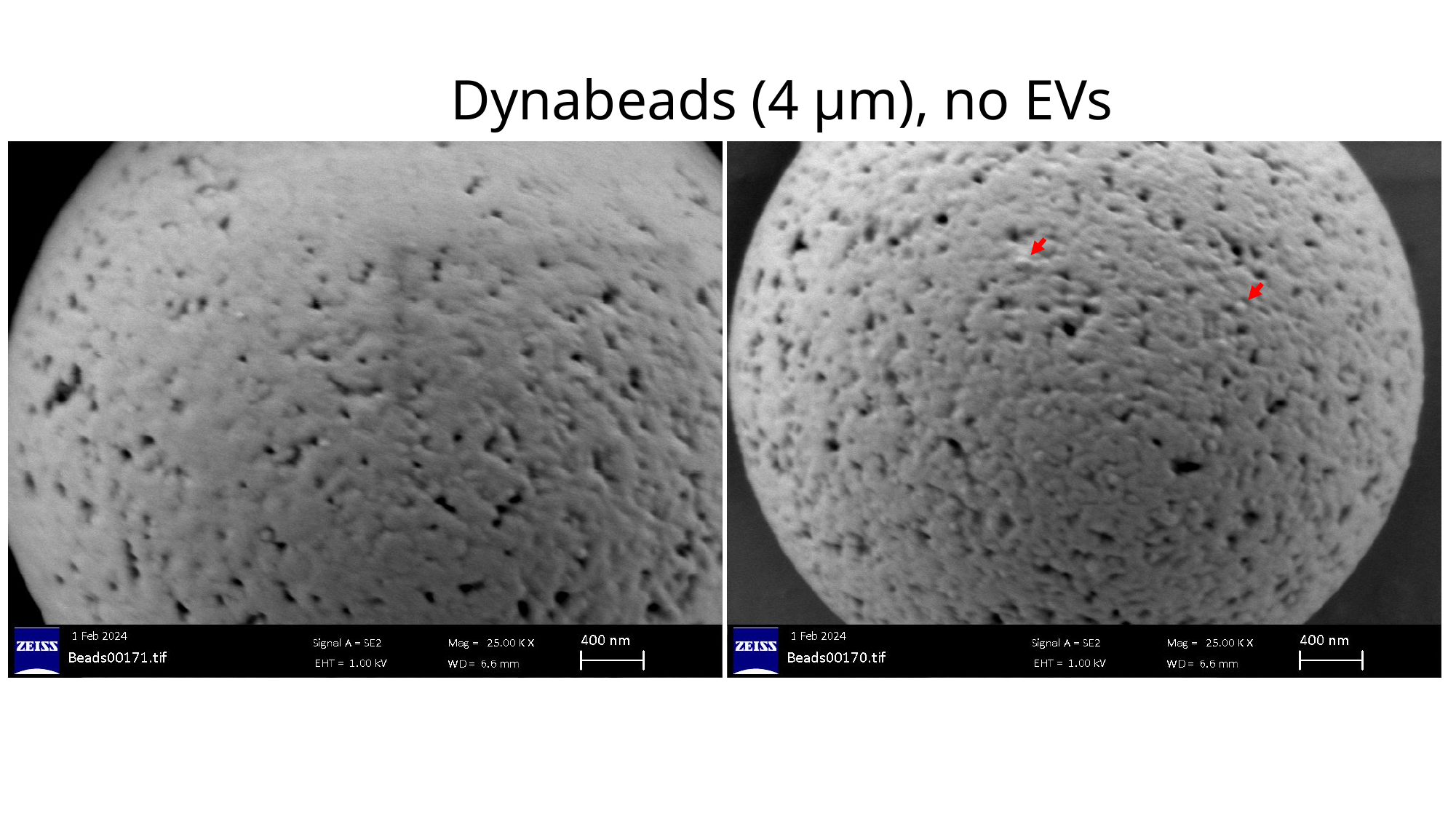

# Dynabeads (4 µm), no EVs

## Slide 7
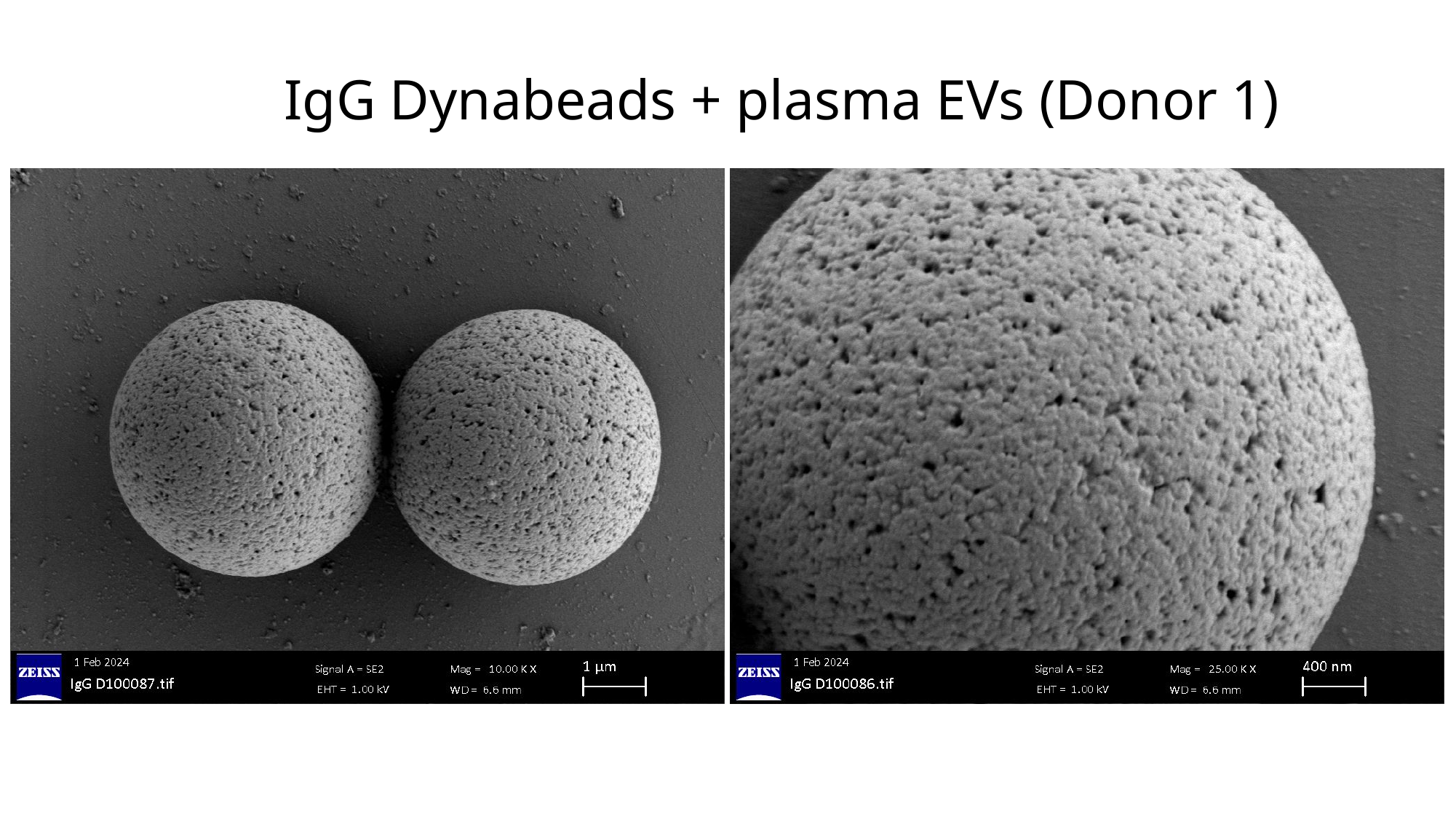

# IgG Dynabeads + plasma EVs (Donor 1)

## Slide 8
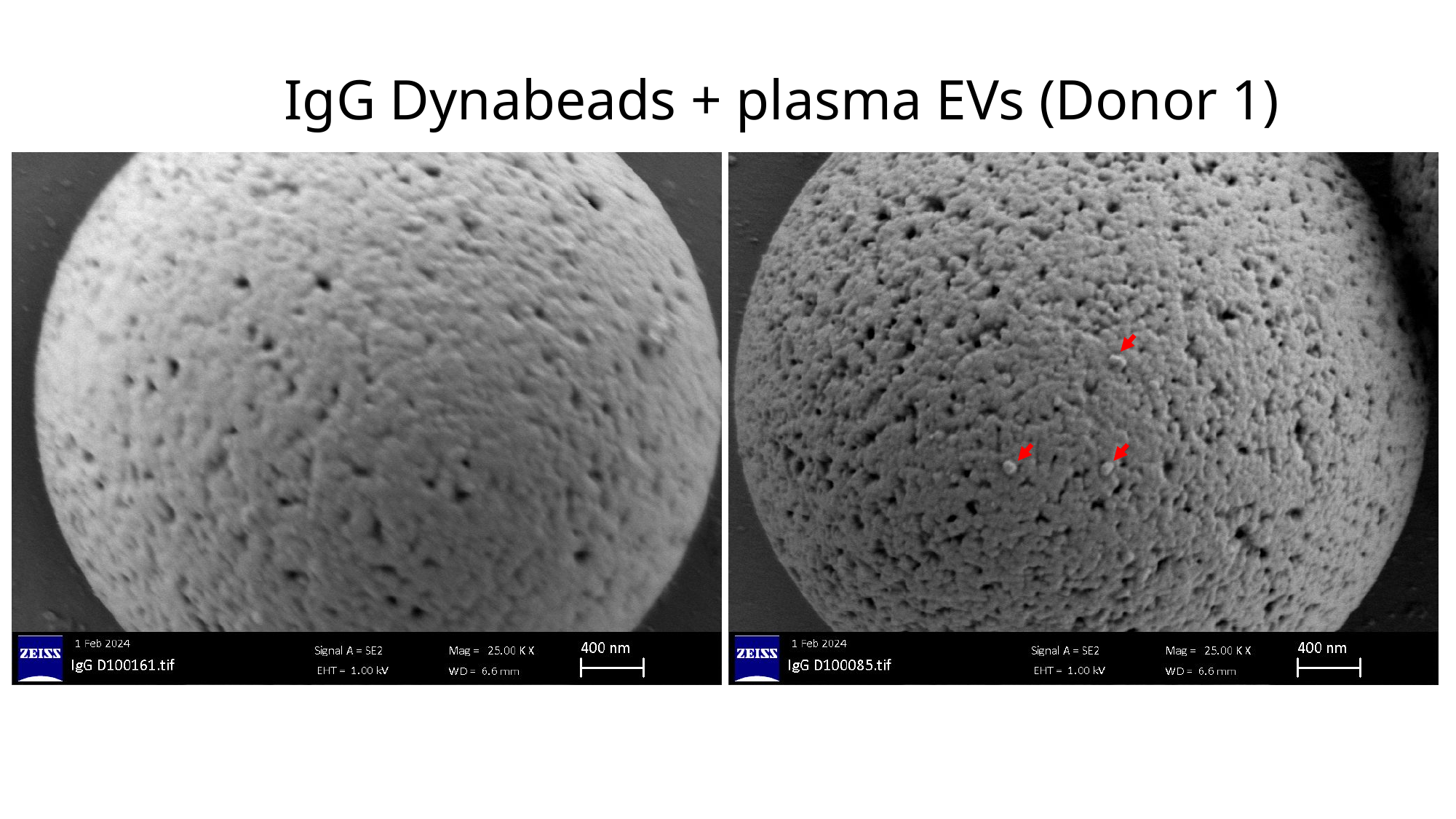

# IgG Dynabeads + plasma EVs (Donor 1)

## Slide 9
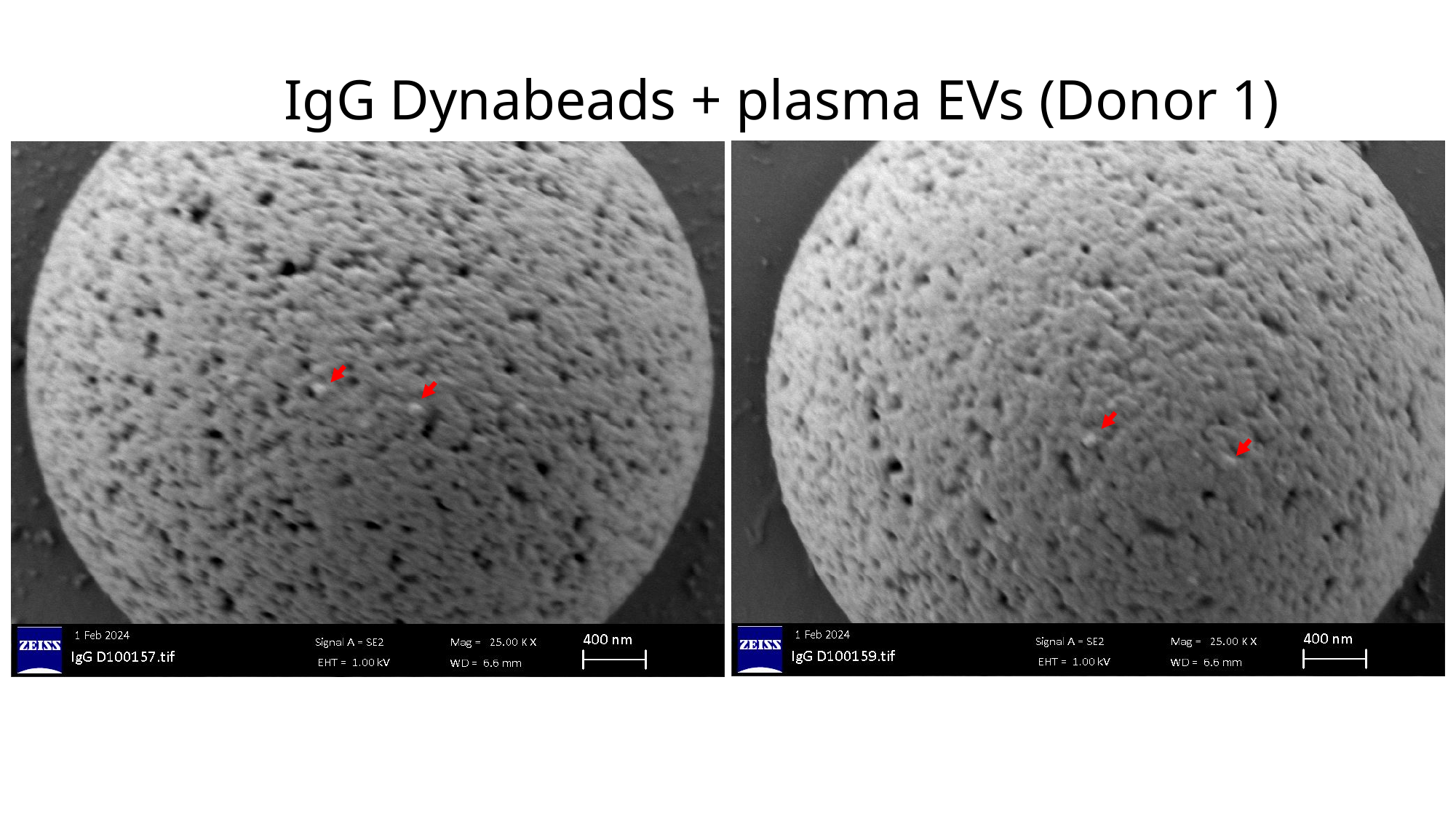

# IgG Dynabeads + plasma EVs (Donor 1)

## Slide 10
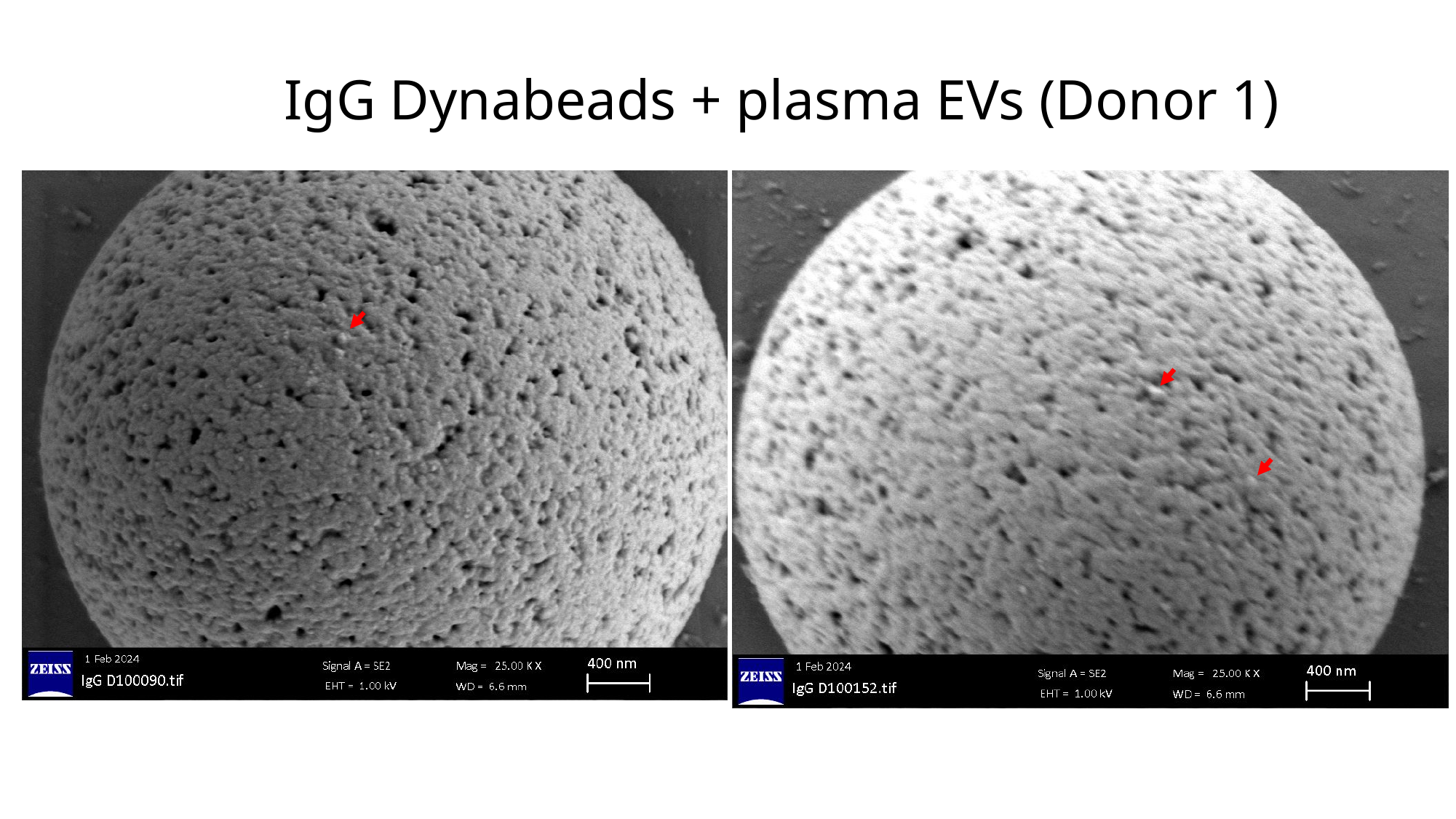

# IgG Dynabeads + plasma EVs (Donor 1)

## Slide 11
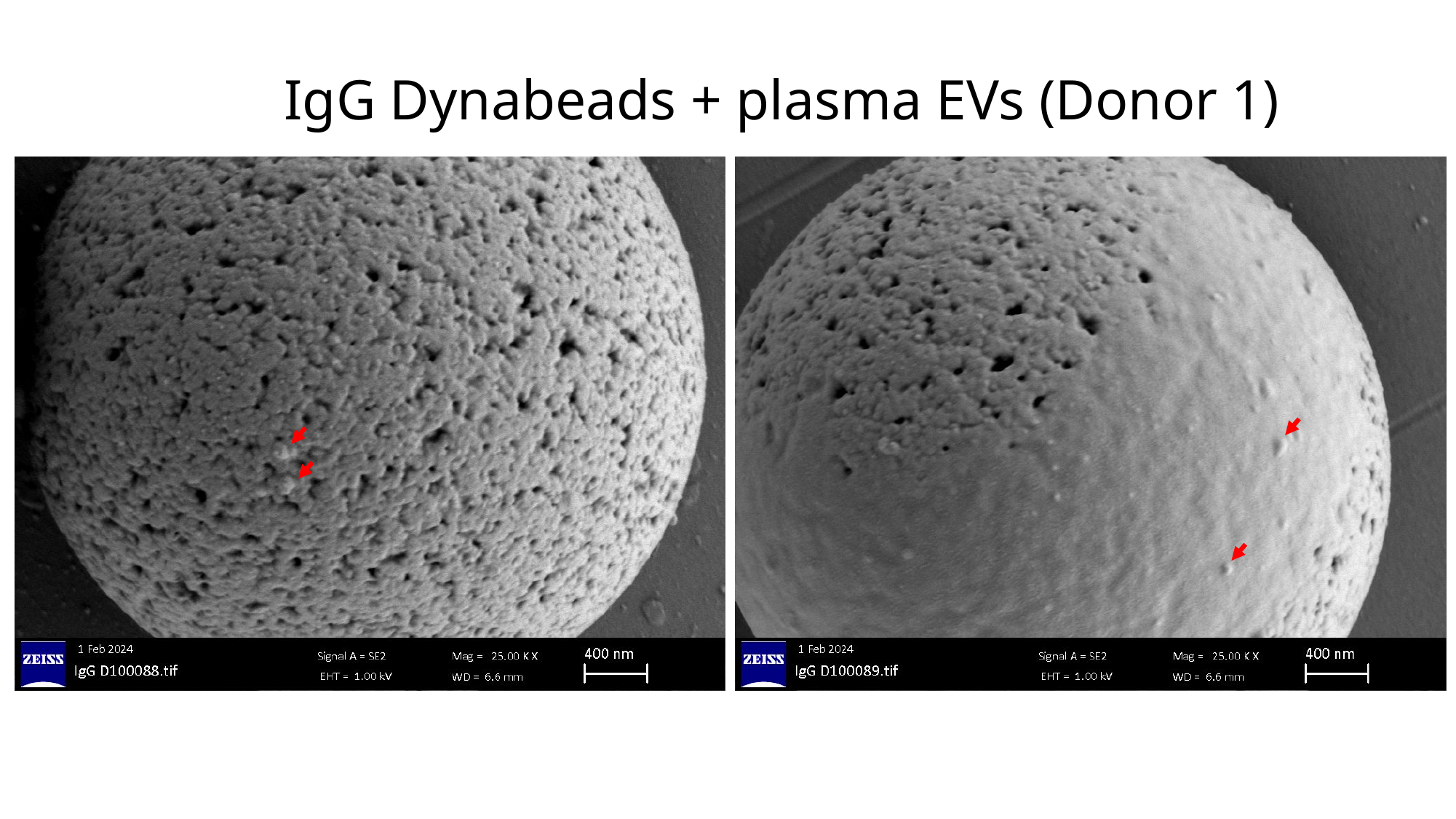

# IgG Dynabeads + plasma EVs (Donor 1)

## Slide 12
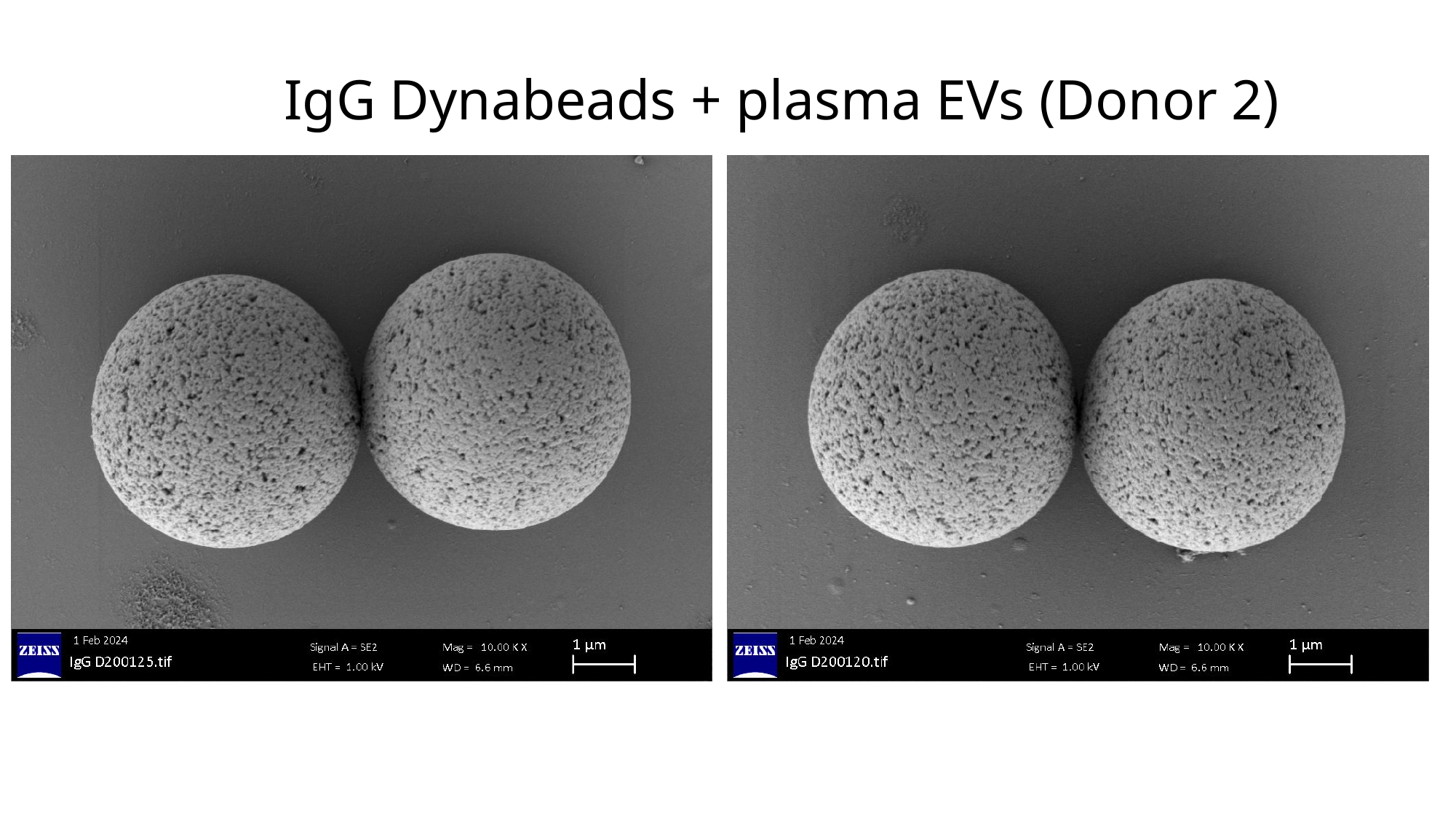

# IgG Dynabeads + plasma EVs (Donor 2)

## Slide 13
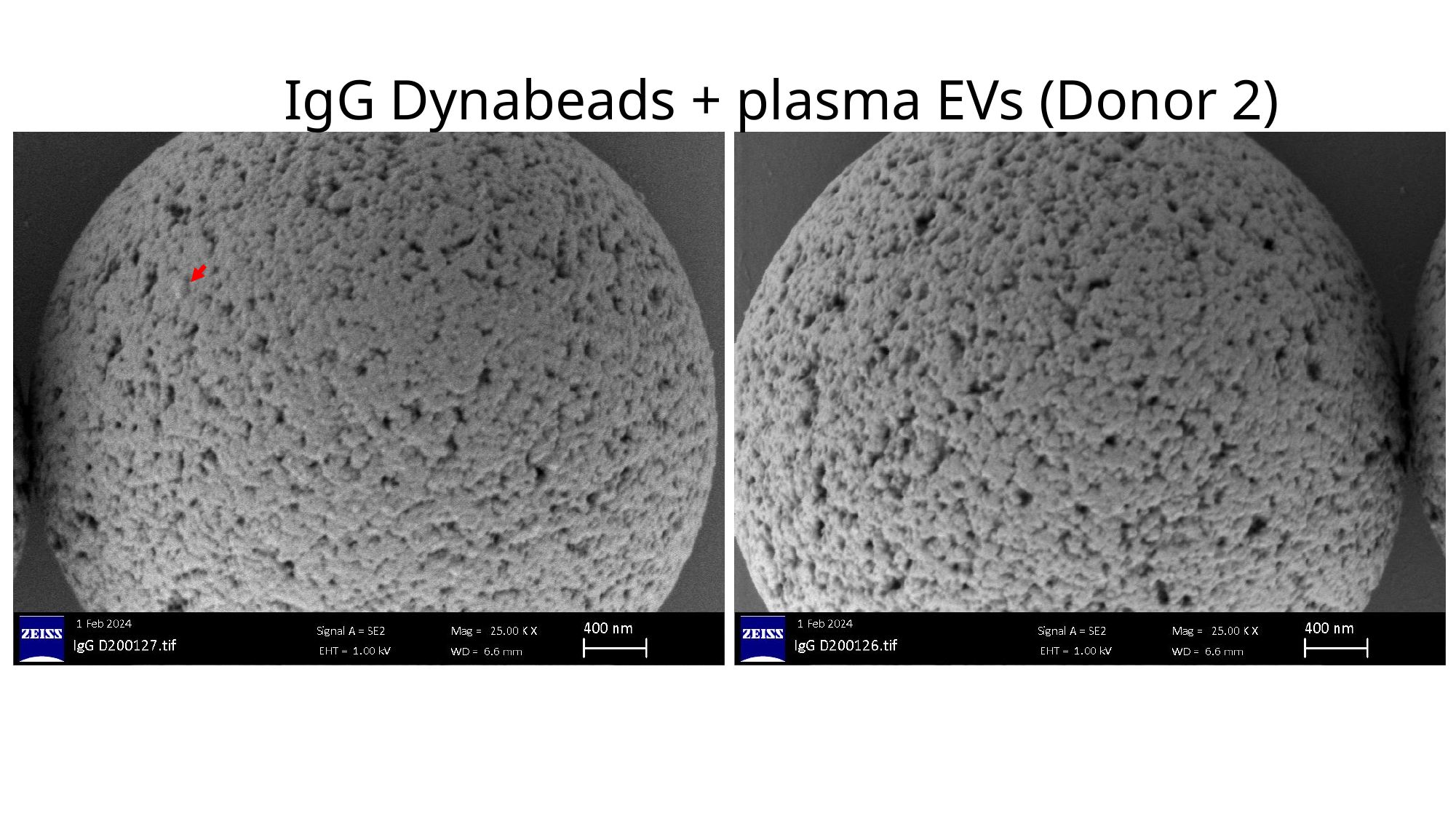

# IgG Dynabeads + plasma EVs (Donor 2)

## Slide 14
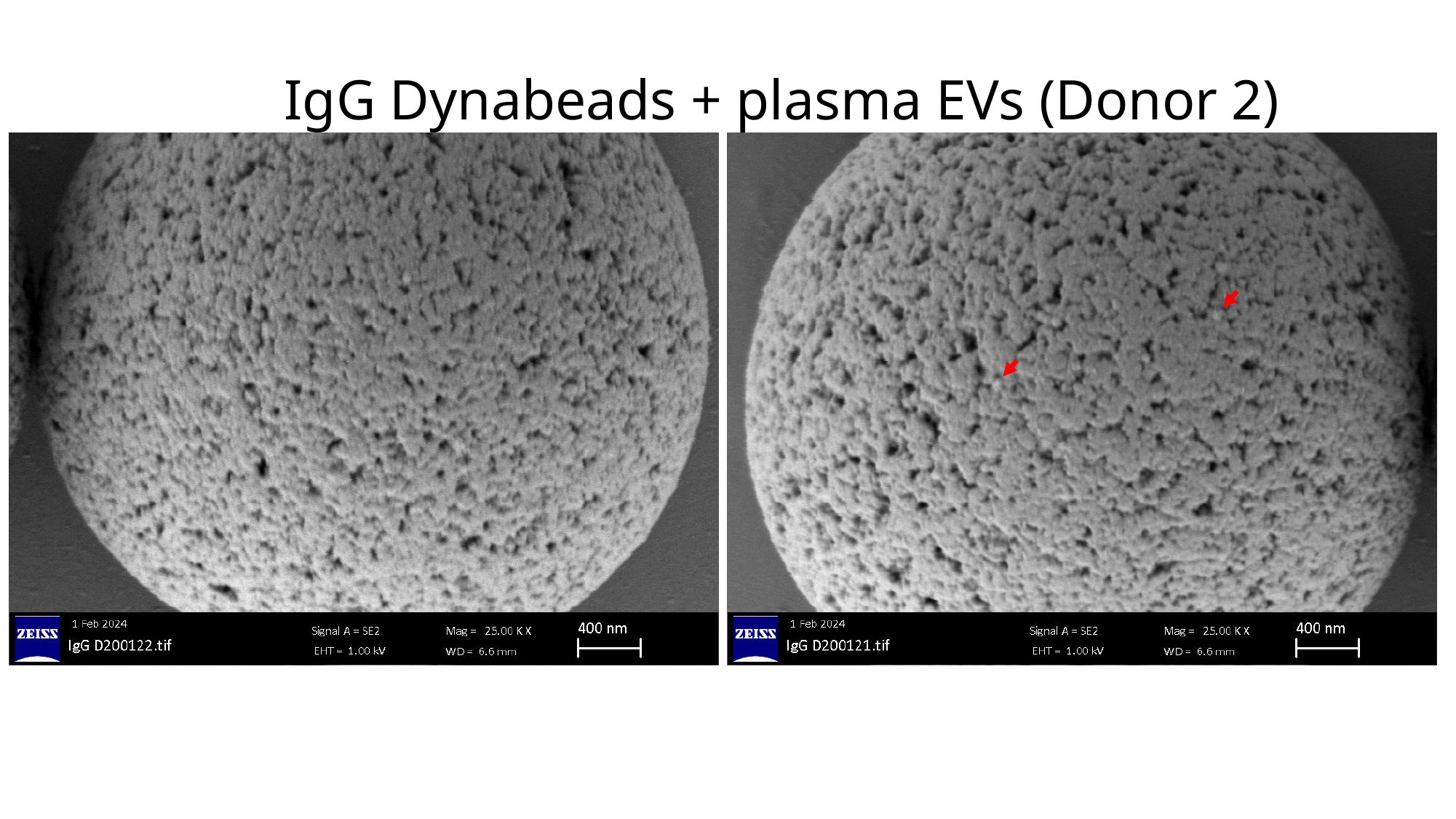

# IgG Dynabeads + plasma EVs (Donor 2)

## Slide 15
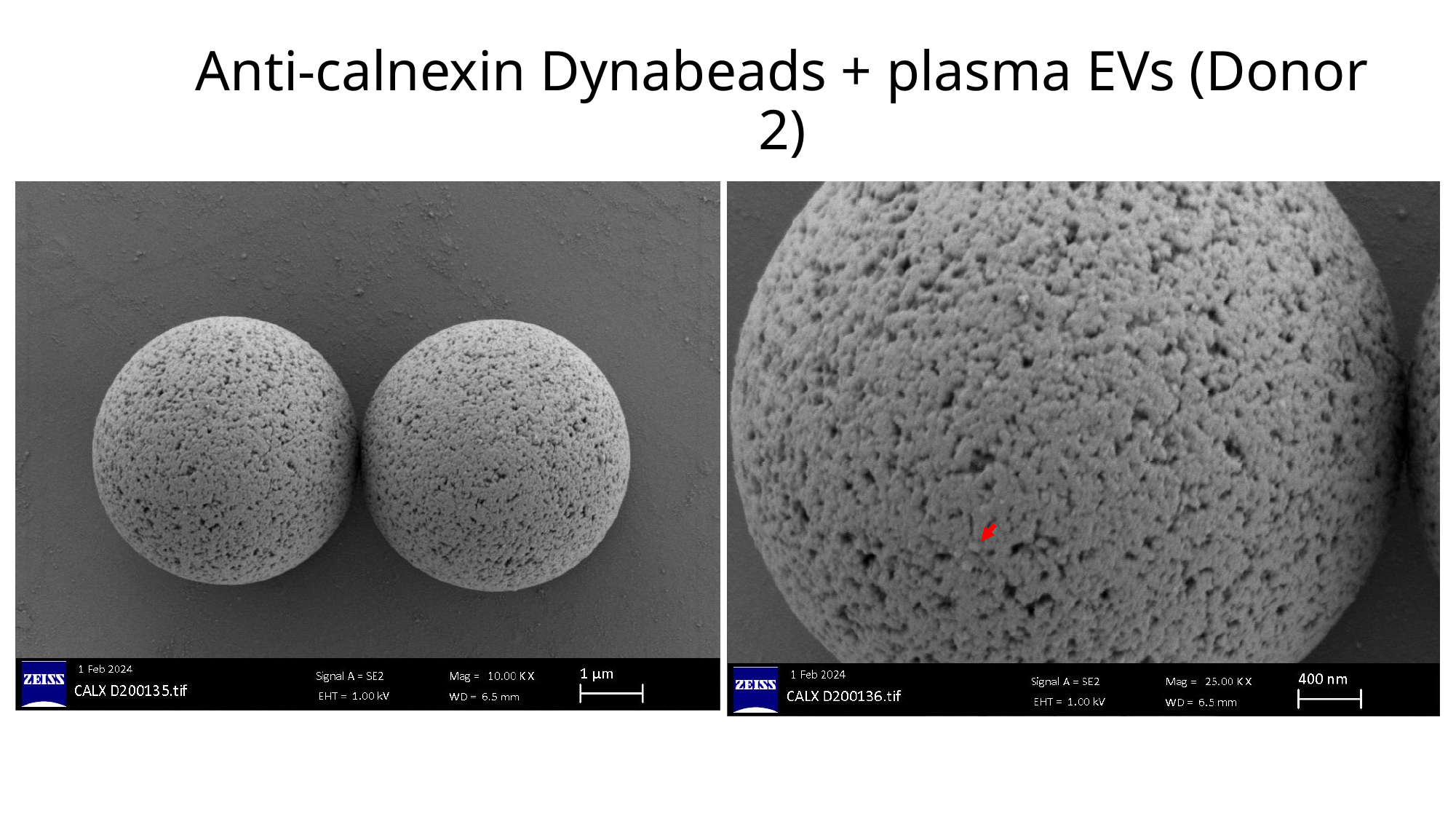

# Anti-calnexin Dynabeads + plasma EVs (Donor 2)

## Slide 16
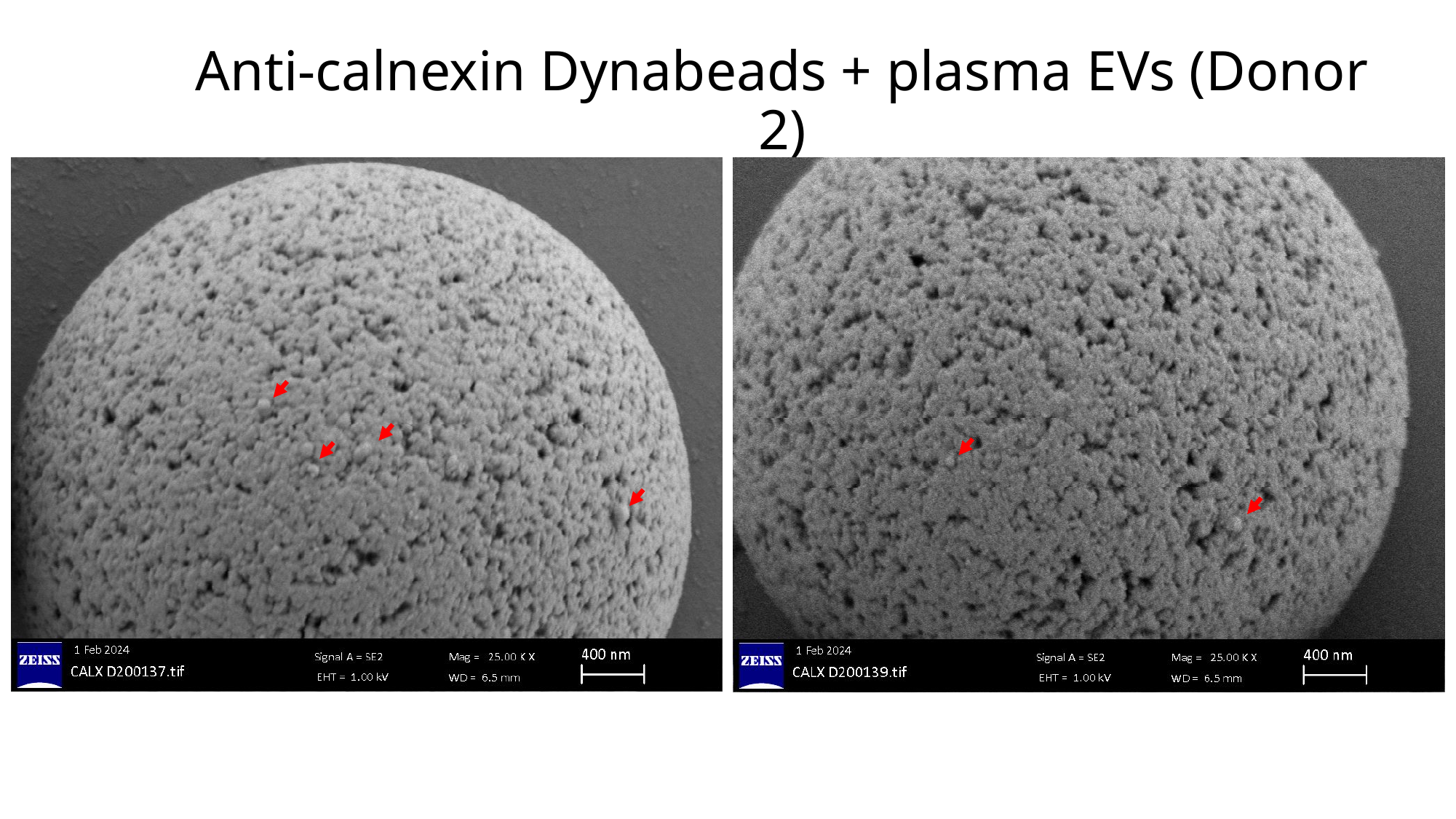

# Anti-calnexin Dynabeads + plasma EVs (Donor 2)

## Slide 17
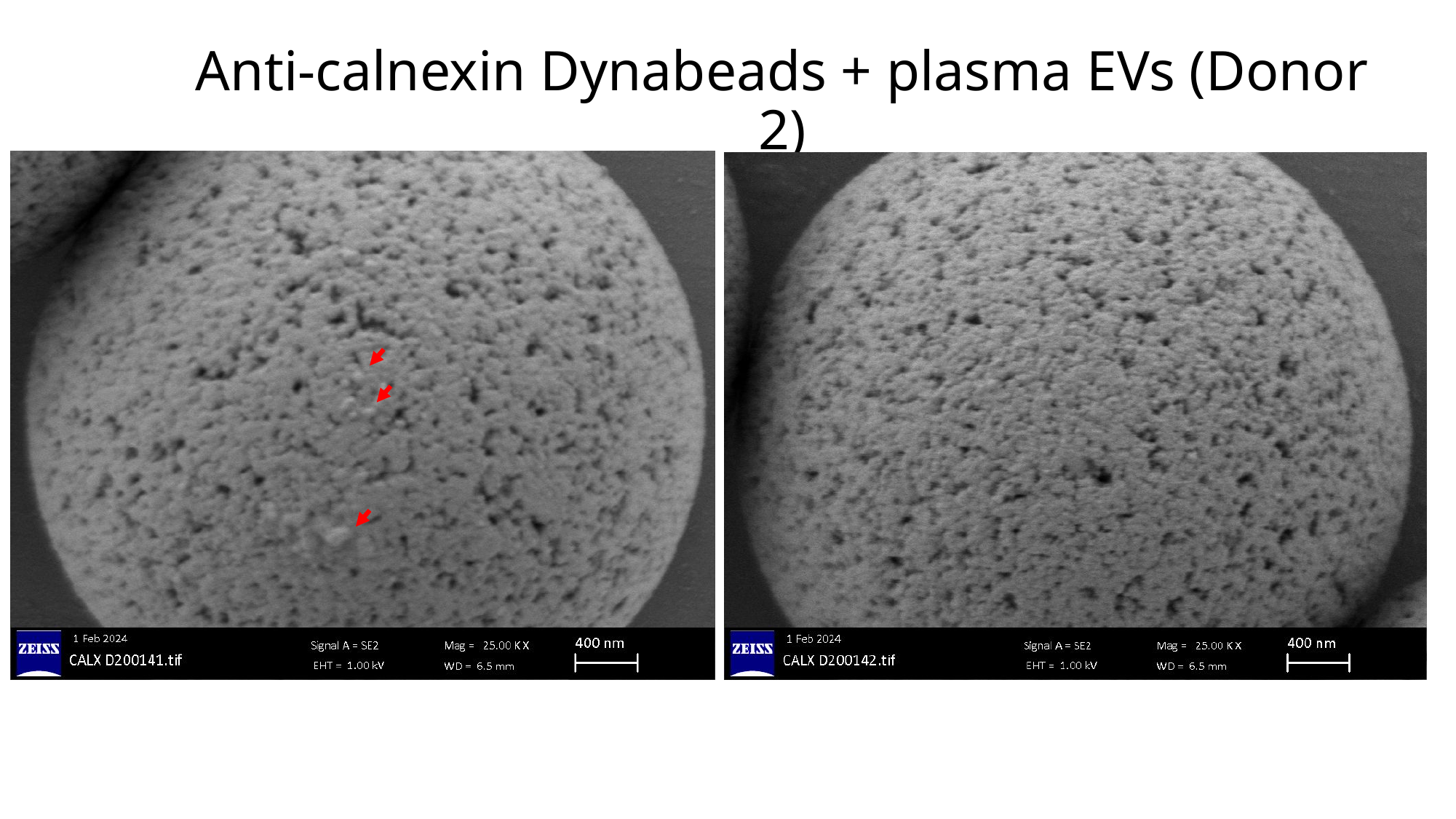

# Anti-calnexin Dynabeads + plasma EVs (Donor 2)

## Slide 18
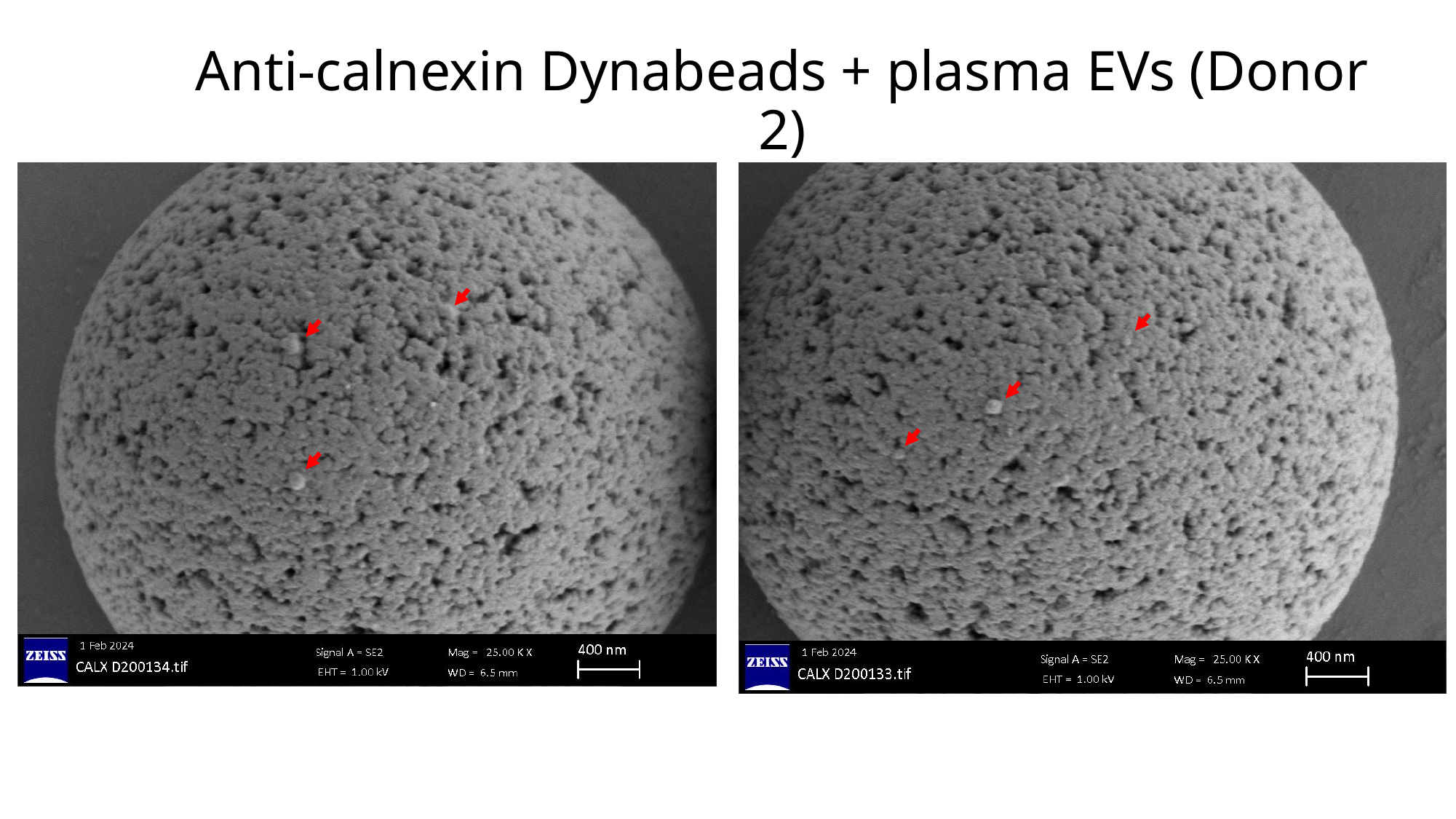

# Anti-calnexin Dynabeads + plasma EVs (Donor 2)

## Slide 19
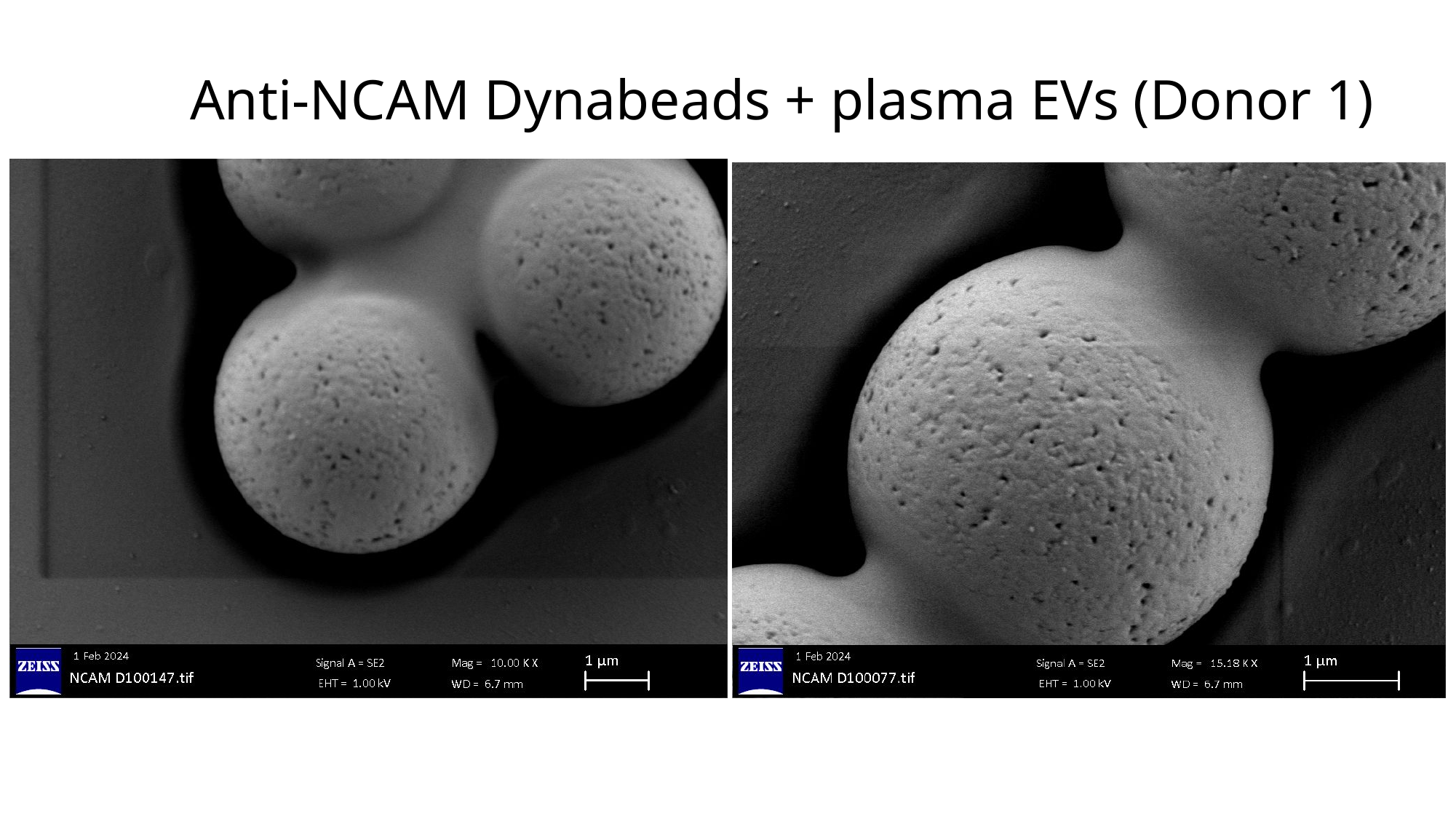

# Anti-NCAM Dynabeads + plasma EVs (Donor 1)

## Slide 20
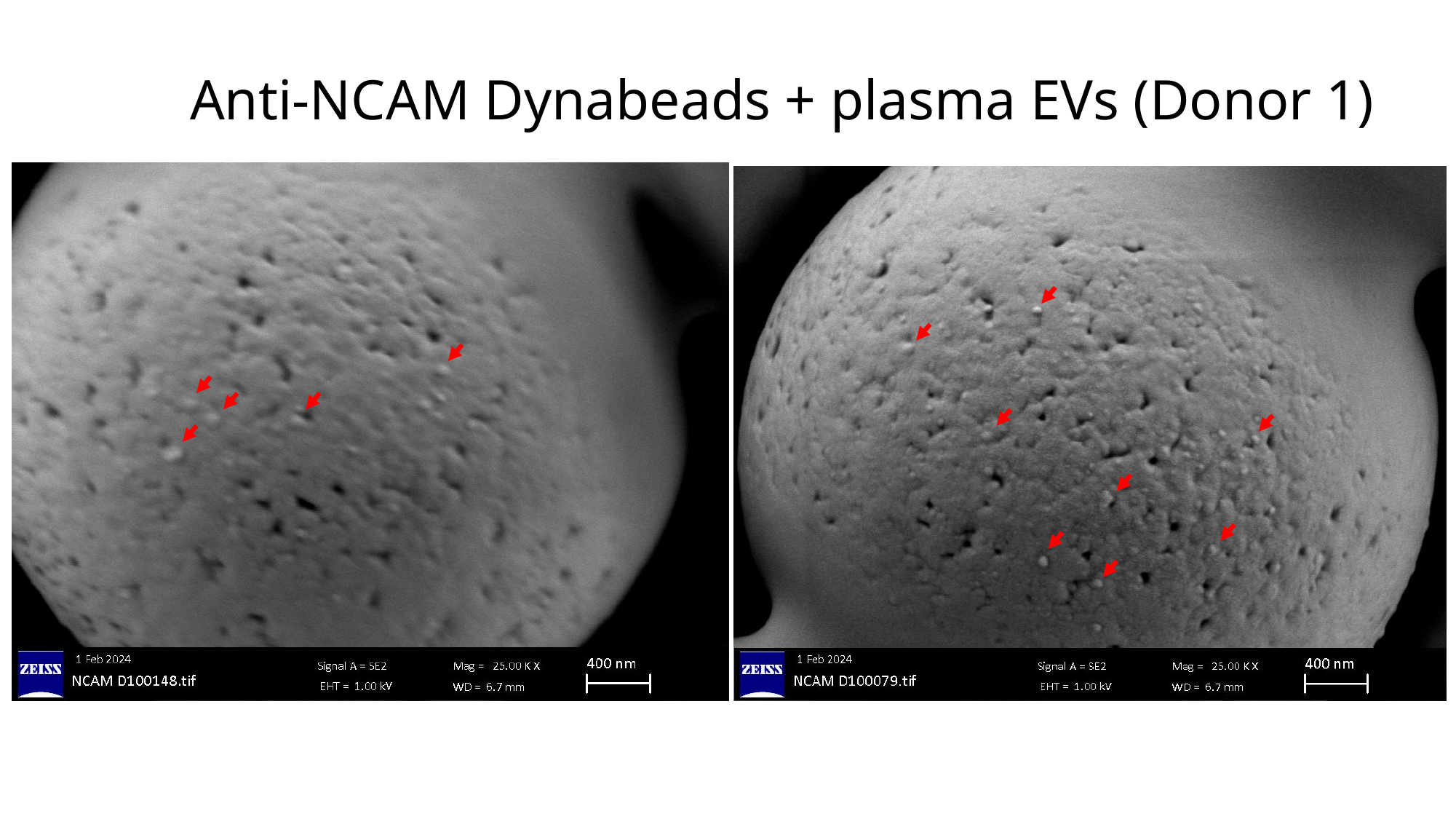

# Anti-NCAM Dynabeads + plasma EVs (Donor 1)

## Slide 21
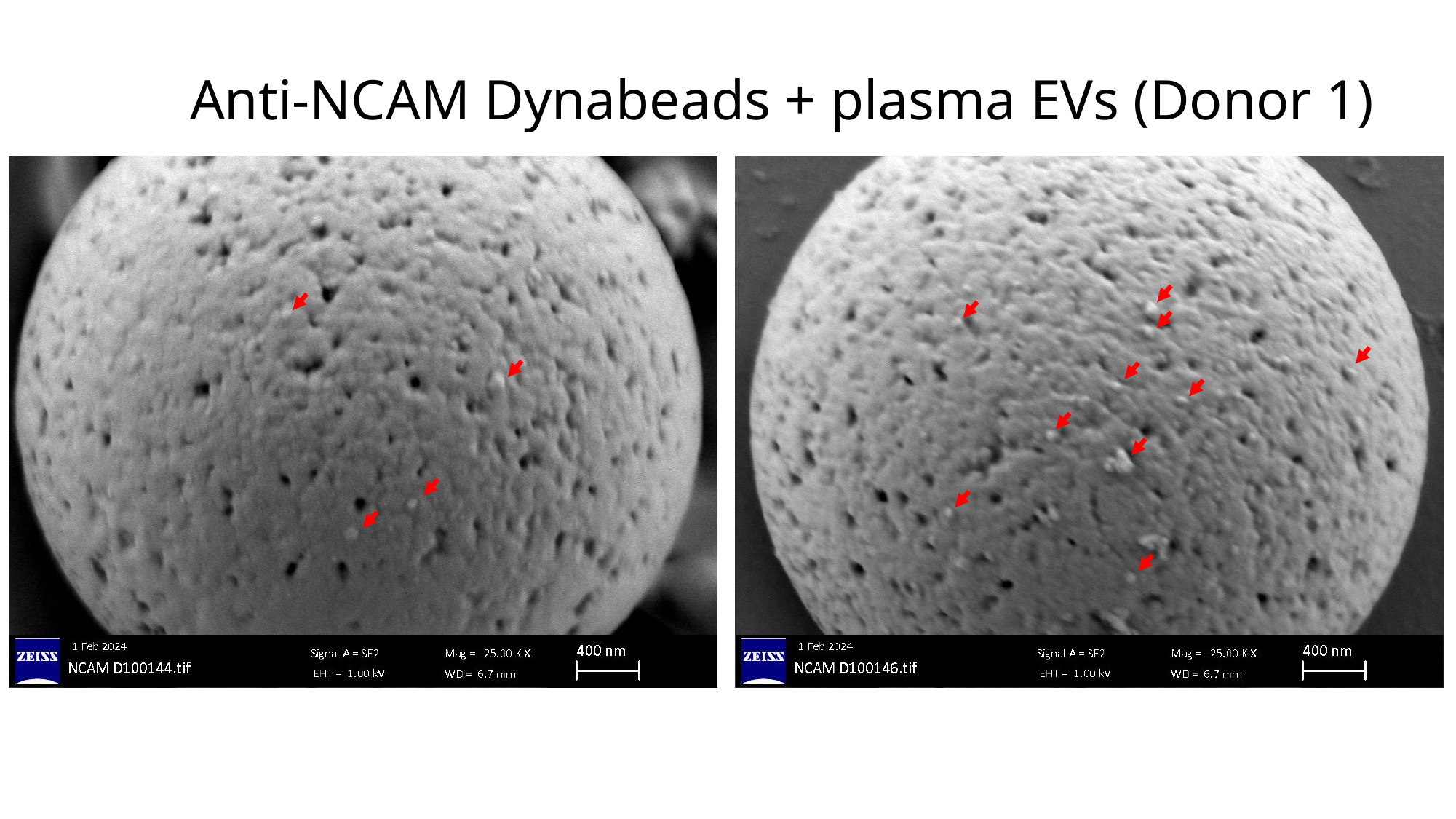

# Anti-NCAM Dynabeads + plasma EVs (Donor 1)

## Slide 22
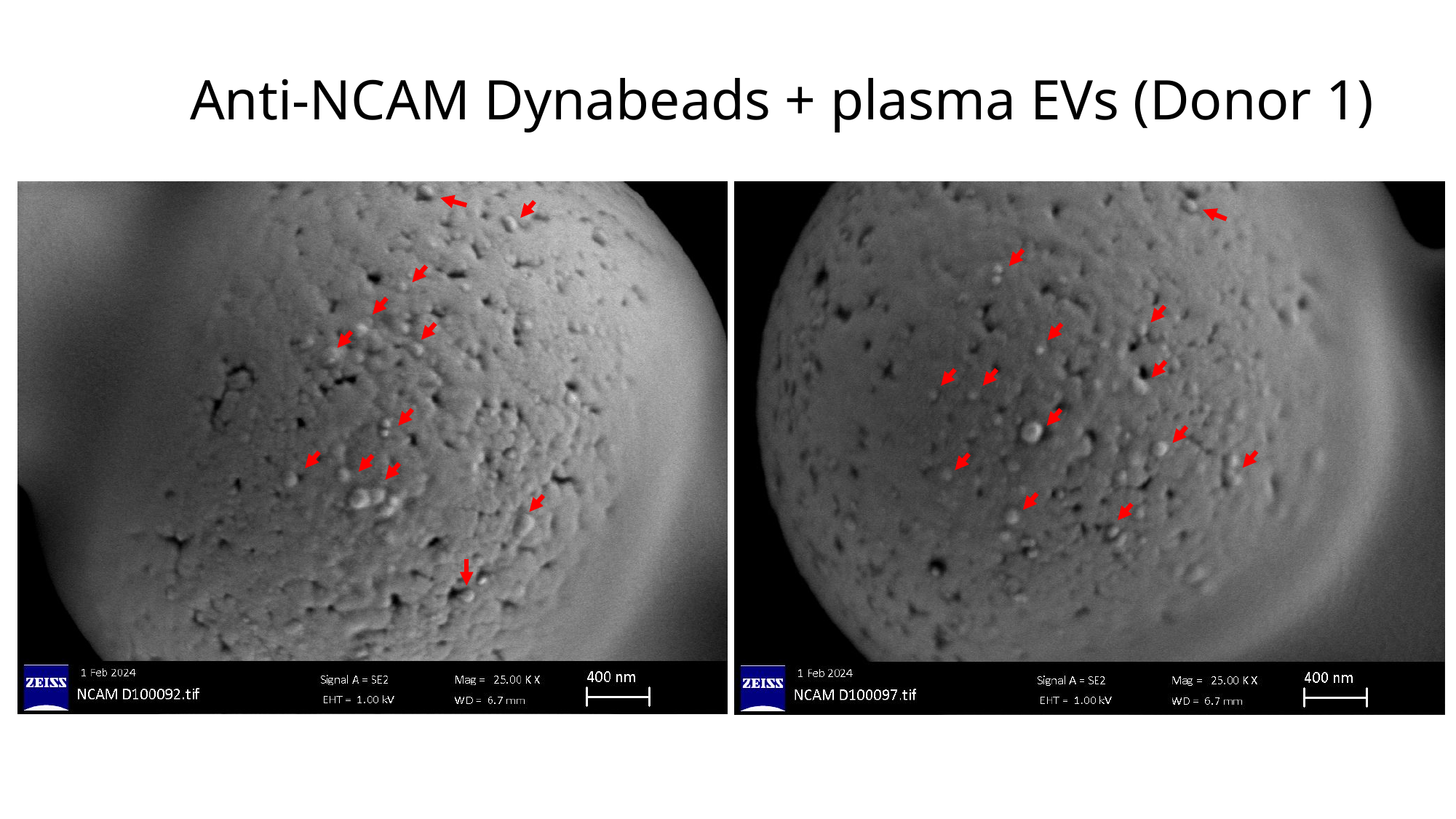

# Anti-NCAM Dynabeads + plasma EVs (Donor 1)

## Slide 23
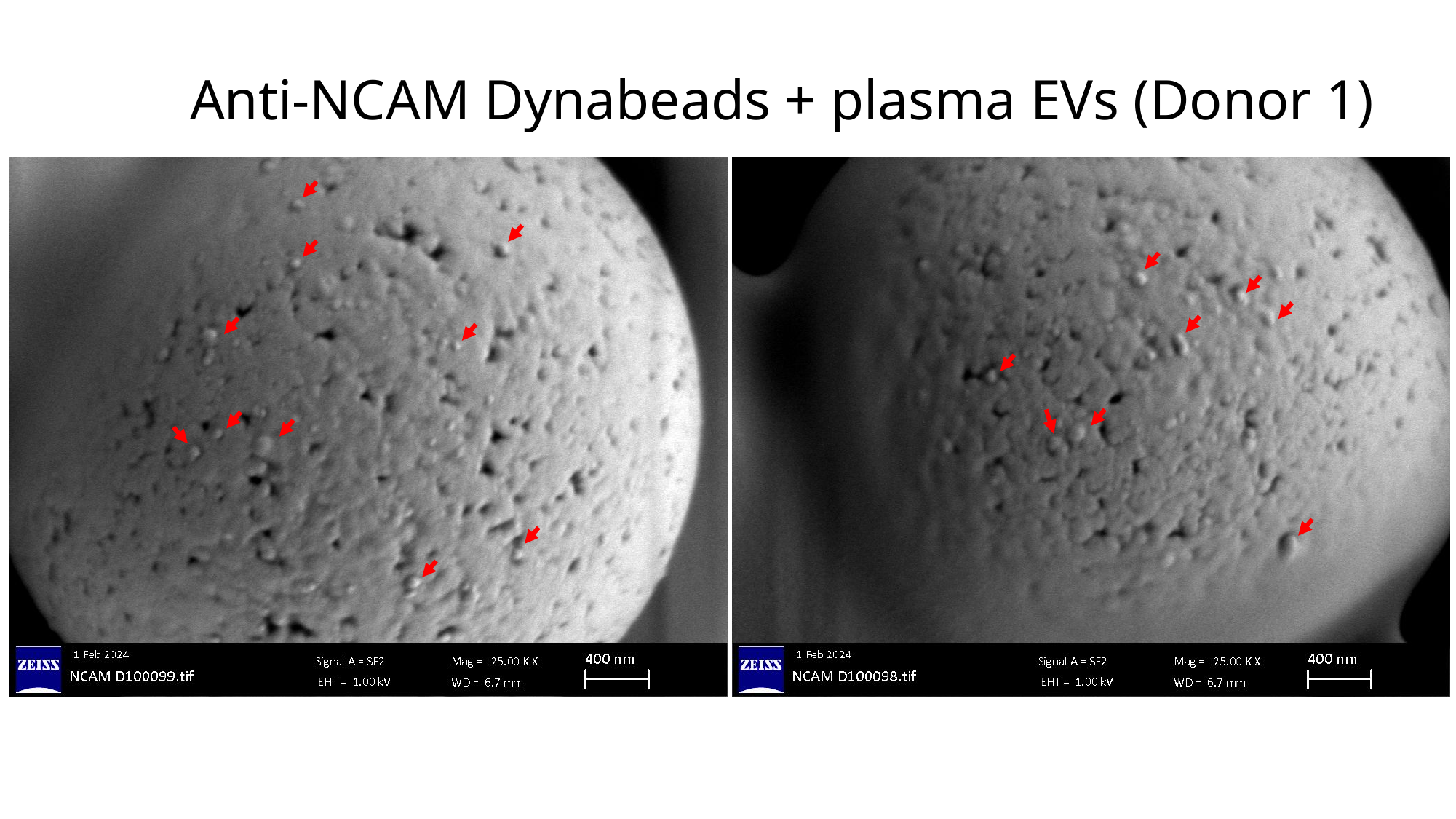

# Anti-NCAM Dynabeads + plasma EVs (Donor 1)

## Slide 24
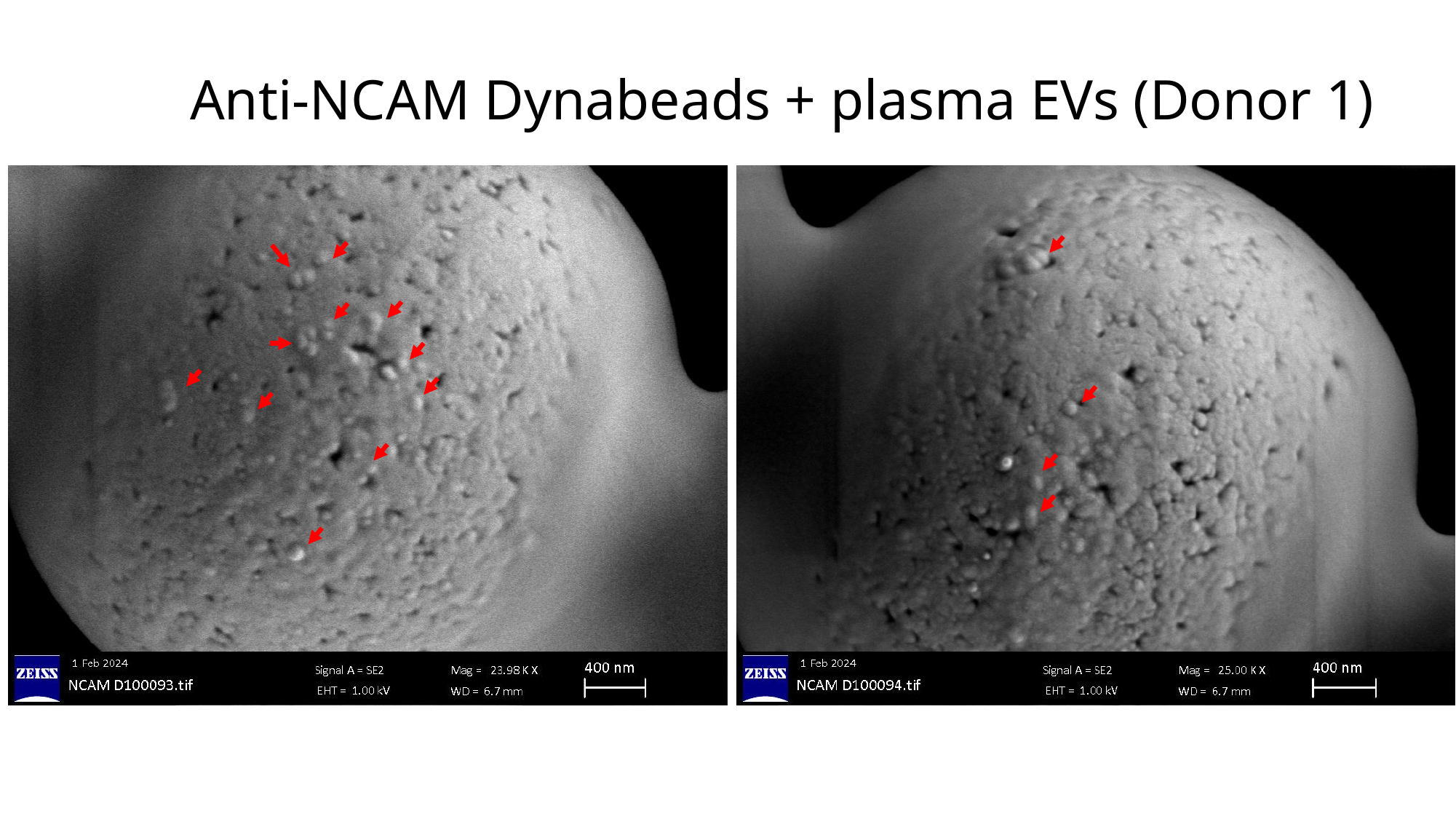

# Anti-NCAM Dynabeads + plasma EVs (Donor 1)

## Slide 25
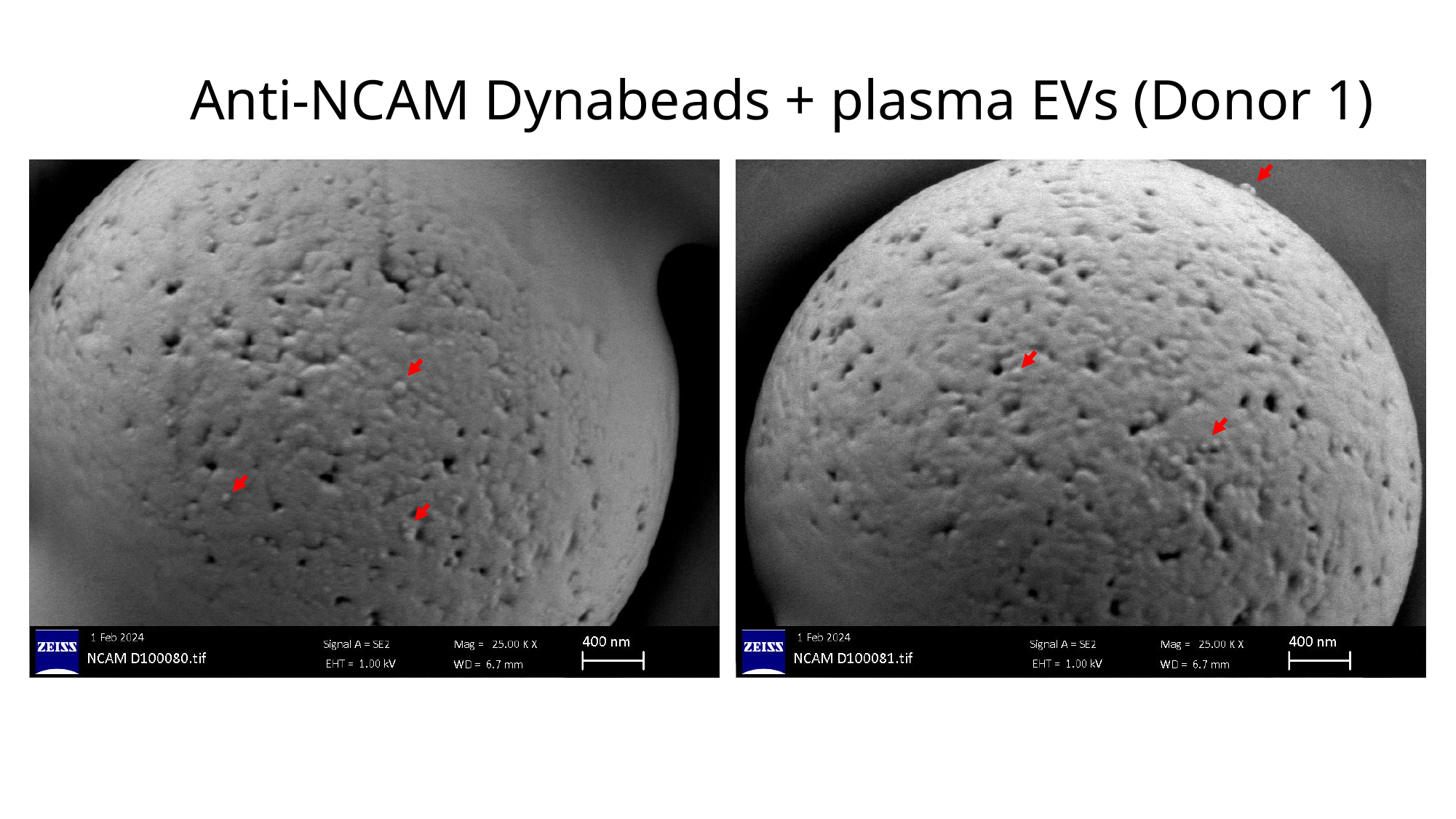

# Anti-NCAM Dynabeads + plasma EVs (Donor 1)

## Slide 26
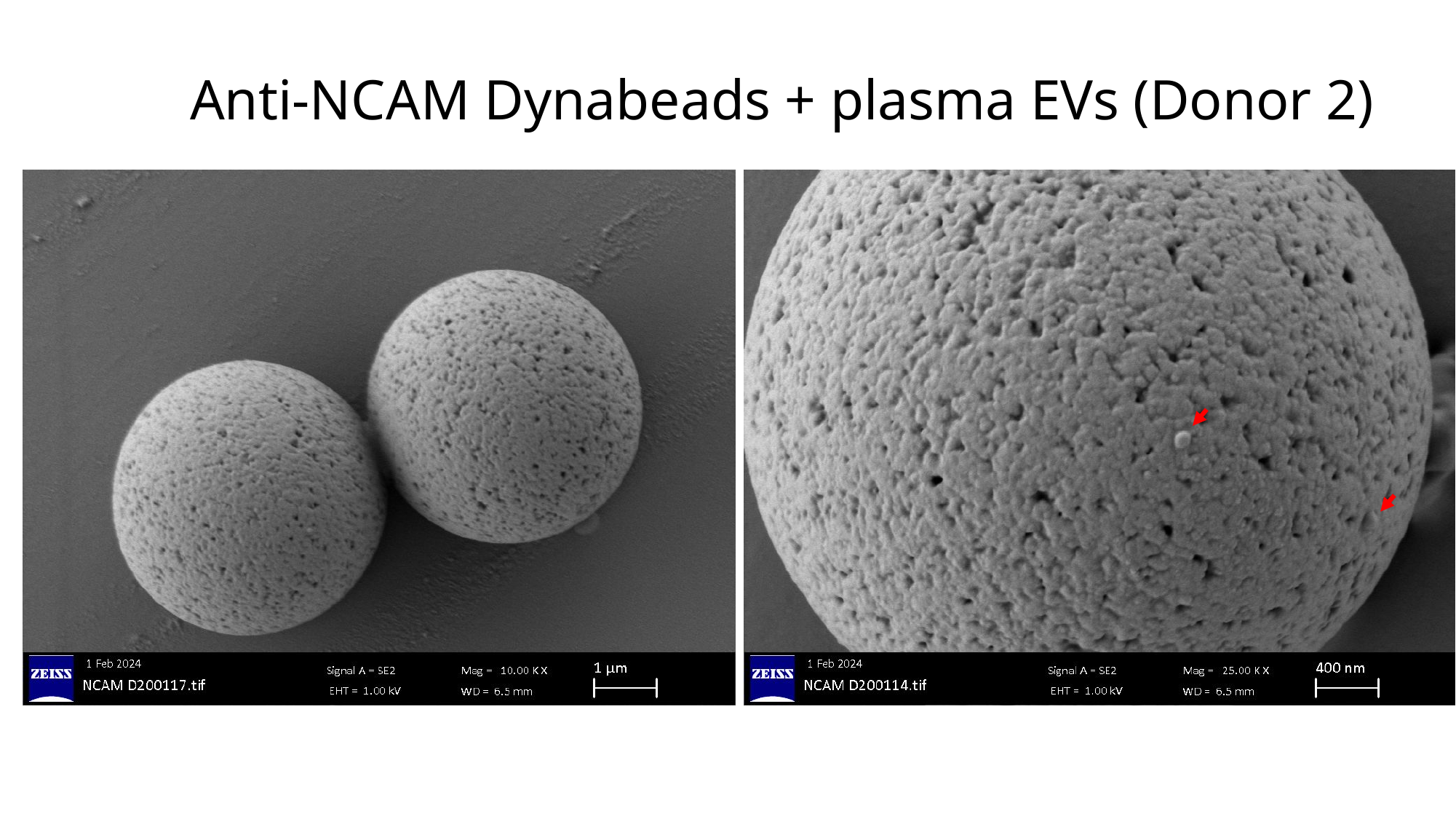

# Anti-NCAM Dynabeads + plasma EVs (Donor 2)

## Slide 27
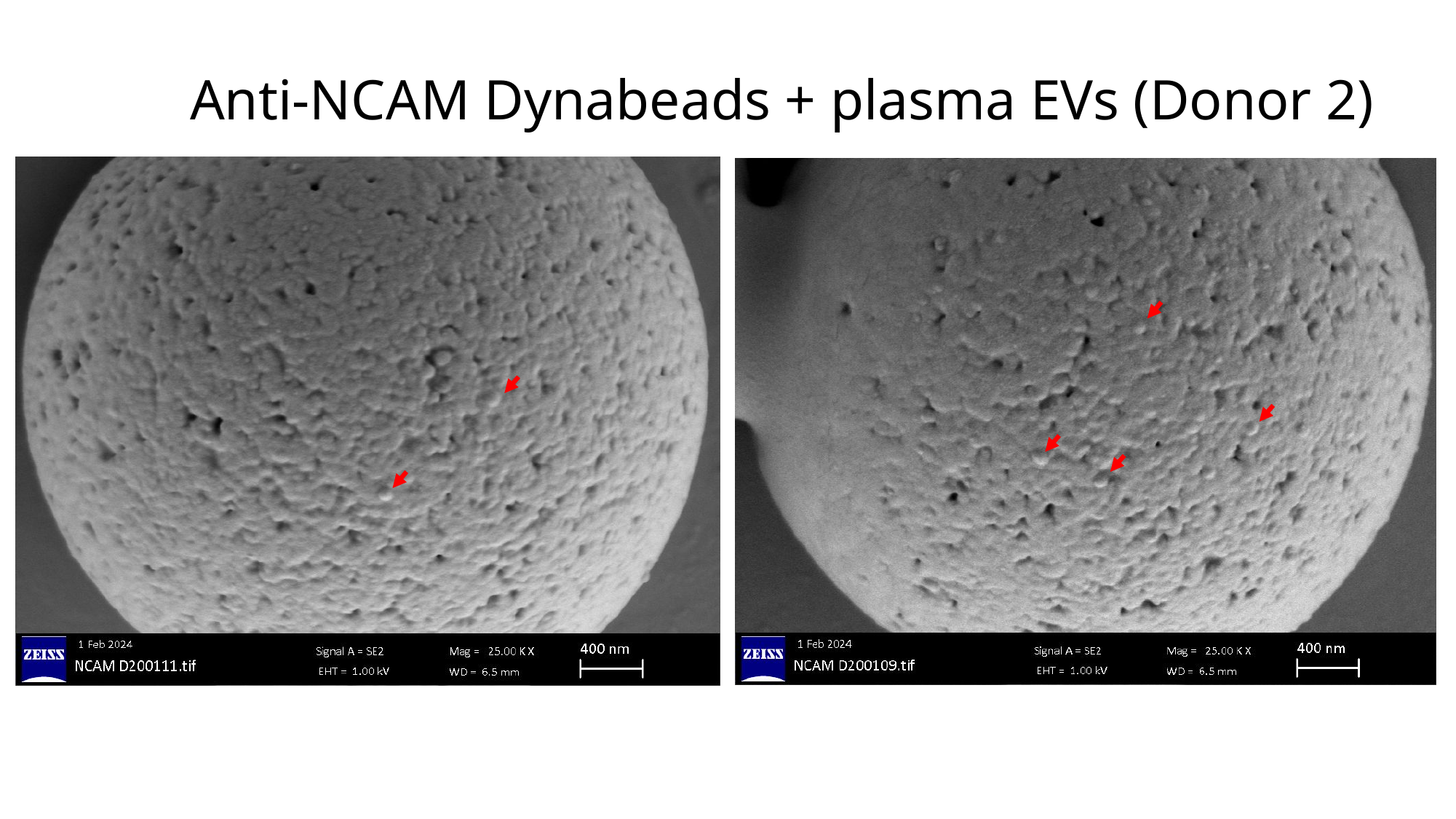

# Anti-NCAM Dynabeads + plasma EVs (Donor 2)

## Slide 28
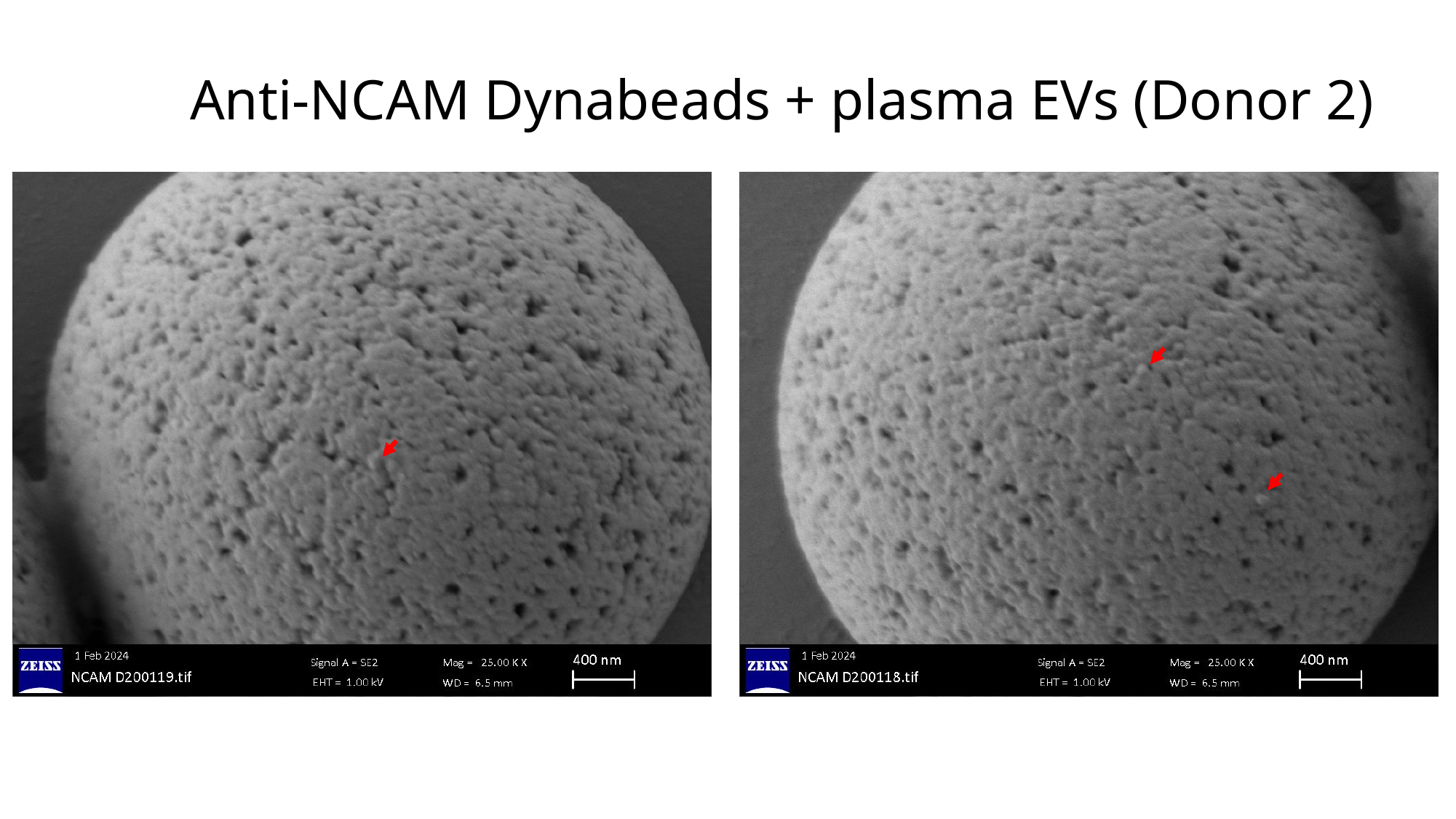

# Anti-NCAM Dynabeads + plasma EVs (Donor 2)

## Slide 29
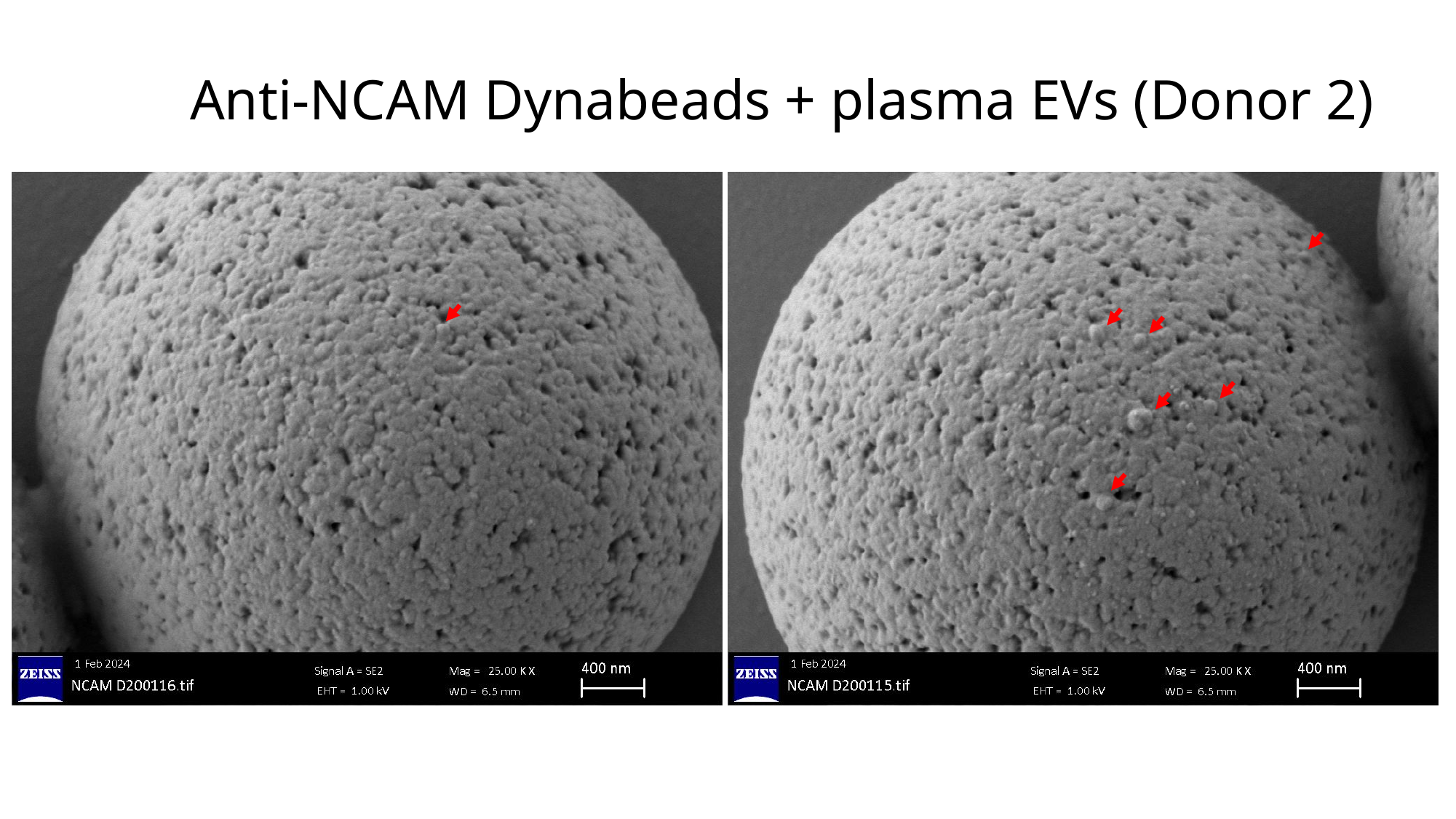

# Anti-NCAM Dynabeads + plasma EVs (Donor 2)

## Slide 30
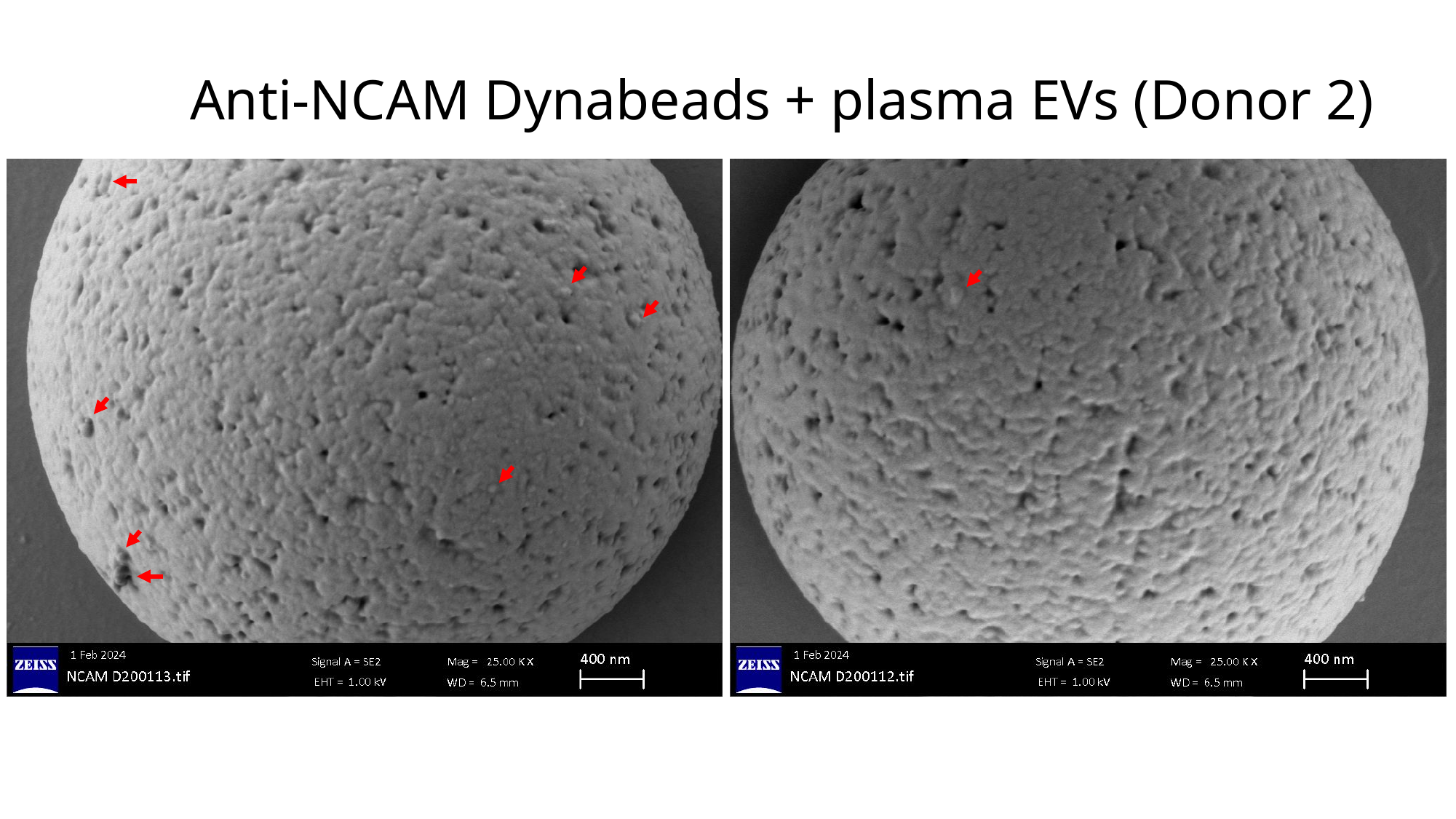

# Anti-NCAM Dynabeads + plasma EVs (Donor 2)

## Slide 31
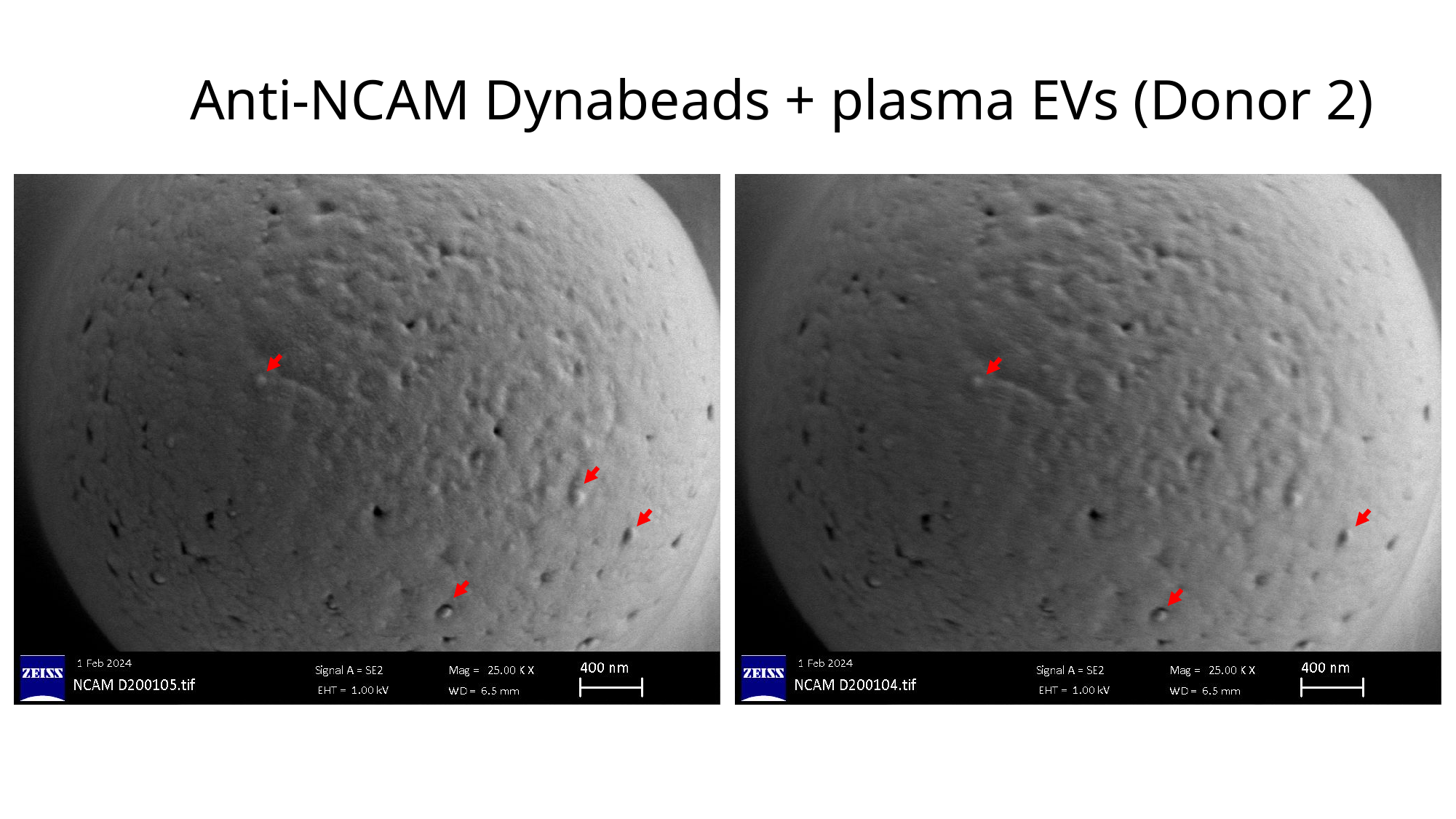

# Anti-NCAM Dynabeads + plasma EVs (Donor 2)
